# Research on Injury Disparities: A Scoping Review

**DOI:** 10.1089/heq.2019.0044

**Published:** 2019-10-17

**Authors:** Megan Moore, Kelsey M. Conrick, Molly Fuentes, Ali Rowhani-Rahbar, Janessa M. Graves, Divya Patil, Madeline Herrenkohl, Brianna Mills, Frederick P. Rivara, Beth Ebel, Monica S. Vavilala

**Affiliations:** ^1^ School of Social Work, University of Washington, Seattle, Washington.; ^2^ Harborview Injury Prevention and Research Center, University of Washington, Seattle, Washington.; ^3^ Department of Rehabilitation, Seattle Children's Hospital, Seattle, Washington.; ^4^ Department of Epidemiology, School of Public Health, University of Washington, Seattle, Washington.; ^5^ College of Nursing, Washington State University, Spokane, Washington.; ^6^ Department of Pediatrics, University of Washington, Seattle, Washington.; ^7^ Department of Anesthesiology and Pain Medicine, University of Washington, Seattle, Washington.

**Keywords:** health equity, health disparities, injury disparities, trauma disparities, violence disparities

## Abstract

**Background:** Research on disparities in traumatic injury has not been well characterized, limiting understanding of gaps in research and development of successful interventions. We conducted a scoping review to identify and synthesize research on disparities in intentional and unintentional traumatic injuries.

**Methods:** The review was guided by PRISMA Extension for Scoping Reviews. PubMed, PsycINFO, Web of Science, and CINAHL and systematic reviews from 2007 to 2017 were searched. Eligible articles were peer reviewed; conducted in the United States; and reported on clearly defined physical trauma and disparity, defined by Cochrane PROGRESS-Plus criteria. One reviewer assessed article titles and a second reviewer validated the inclusion with a random sample. Abstract and full-text review by two reviewers determined final inclusion.

**Results:** Of 7382 unique articles screened, 653 articles were included; inter-rater agreement was high (*K*=0.995). Studies reported on disparities in the acute hospital setting (104) or postacute/rehabilitation (86), with fewer focused on prevention (57) and policy development (6). Research methods used were quantitative (593) with 25 intervention studies, qualitative (45), qualitative/quantitative (7), and community-based participatory research (8). Age ranges of included studies were all ages (124), adults (318), pediatric/youth/adolescents (172), and older adults (40). Racial disparities were most commonly measured (439 studies); 38 created a white/nonwhite binary. Other commonly measured disparities were place of residence (122), insurance (111 studies), gender (89), age (75), and socioeconomic status measures (61). Disparities were noted in all of the categories. Studies commonly aggregated all types of traumatic injuries (129) or all types of violence (105).

**Conclusions:** The extant injury literature lacks research on prevention and policy to address disparities. Many studies aggregated types of trauma and patient groups, preventing an understanding of distinctions between groups and potential interventions. Intervention and community-based research strategies were limited. Future research can better specify measurement of understudied equity categories, trauma types and intent, and racial groups.

## Introduction

Injuries are a major public health concern. Physical injuries, including those that result from violence, self-harm, and unintentional trauma, are a leading cause of death and disability in the United States.^1^ Injuries cause ∼200,000 deaths, and 30 million individuals are treated for injuries in hospitals and emergency departments each year.^2^ The Centers for Disease Control and Prevention estimates that injuries, including violence and unintentional injuries, cost more than $671 billion/year in medical care and lost productivity.^2^

Disparities in injury incidence, treatment, and outcomes have been documented^3–5^ and frequently result from social determinants of health. Therefore, the burden of injury falls disproportionately on communities of color, those who are economically disadvantaged, and those who are geographically isolated.^3–5^ The National Academies of Sciences, Engineering, and Medicine has called for further research to inform both the mechanisms of and interventions to address health disparities and inequities.^6^ To our knowledge, no existing reviews have described the injury disparity literature at large. This gap prevents an understanding of research needs to guide practitioners and researchers in identifying mechanisms that result in disparities and hinders the development of successful interventions to reduce injury-related disparities and inequities.

We conducted a scoping review of the literature to identify, assess, and categorize existing injury disparity research. We addressed the following research questions: (1) What are the types (and quality) of studies on injury disparities? (2) What equity-related data elements are reported and how specified are these elements? (3) Which populations and disparities are included and excluded in existing research? We chose a scoping review due to the large-scale and exploratory nature of these questions and because our objectives were to identify key trends and gaps in research on injury disparities.^7,8^ Results of this review may be of particular interest to health care and community providers serving injured patients and to researchers and policy makers interested in improving injury science to develop solutions to achieve injury-related health equity across the life span.

## Methods

### Review design

We conducted a scoping review of the literature using the PRISMA Extension for Scoping Reviews.^7^ We engaged two health sciences librarians to assist in development of the search strategy. This study did not require institutional review board approval.

### Search methods

We searched the following databases for peer-reviewed research and systematic reviews of physical injury and disparity published from January 1, 2007, to December 31, 2017: PubMed, PsycINFO, Web of Science, and CINAHL. We used database-specific terms as appropriate. Systematic reviews were hand searched, duplicates removed, and additional articles included. The following is an example of the search strategy from the PsycINFO database: (MH “Healthcare Disparities” OR MH “Health Status Disparities” OR disparity OR disparities OR equit* OR inequit* OR inequalit*) AND (injury OR injuries OR trauma* OR violence OR MH “Violence” OR MH “Wounds and Injuries”). See Supplementary Data for a complete list of search terms.

### Selection criteria

Eligible articles were peer reviewed and published in English between 2007 and 2017 because we were interested in the most recent advances in the field. We also limited articles to those conducted in the United States because mechanisms that lead to disparities and methods for addressing them vary by nation. We included studies that reported on a clearly identified physical injury, including violence and unintentional injury, and disparity. Disparity was defined by Cochrane's Methods for Equity Systematic Reviews PROGRESS-Plus criteria,^9^ which include place of residence, race/ethnicity/culture/language, occupation, gender, religion, education, socioeconomic status (SES), social capital, and personal characteristics associated with discrimination (e.g., age and disability). Studies were excluded if they were editorials, reported on opiate use, or reported on the impact of ancestral or structural trauma (e.g., historical trauma among a cultural group and its effect on individual-level current substance use). Studies of intimate partner violence and child maltreatment were excluded because of existing disparity-focused systematic reviews in these areas.^10–19^

### Data extraction

Two reviewers assessed study titles for initial inclusion; one reviewer assessed all articles and a second reviewer validated the inclusion decision using a random sample of 10% of articles retrieved from the initial search. A kappa statistic was calculated to determine inter-rater reliability. The second round of review included abstract searches of articles included in the first round and was conducted by two reviewers. The third round included a full-text search by two reviewers to assess final inclusion. Next, five members of the research team created and used a prespecified data extraction form. Data extracted included year published, journal and impact factor, disparity type studied (e.g., race), study setting (e.g., prevention and rehabilitation), age of participants (e.g., pediatric), injury type, and study design. Study design was defined using the following categories: quantitative, qualitative, qualitative/quantitative, and community-based participatory research (CBPR). The quantitative category included descriptive and analytic epidemiology, meta-analysis, Delphi process, and intervention studies. The qualitative category included conceptual studies and those using qualitative content analysis of interviews and focus groups. The qualitative/quantitative category included studies using both qualitative analysis and quantitative analysis. The CBPR category included studies in which authors identified the use of CBPR, irrespective of the analytical approach.

### Data synthesis and analysis

Descriptive statistics were recorded for the year of publication, journal of publication and impact factor, disparity studied, study setting, age of participants, and study design. All categories were mutually exclusive, except disparity studied. Due to the heterogeneity of study design and scope of the review, we did not conduct a meta-analysis. Journal impact factors were used as a proxy for rigor. Study design categories were determined by the research team. We used a previously designed framework^20^ for classification of injury when possible; categories included definitions of injury by anatomy, pathological mechanism, etiological mechanism, intent, severity, event, location, and activity. Because these categories can overlap, a hierarchy of classification was developed and agreed upon by three members of the research team to allow for reporting of findings in mutually exclusive categories. Some categories were combined. When studies did not disaggregate injury types or type of violence, they were labeled as such.

## Results

**FIG. 1.**
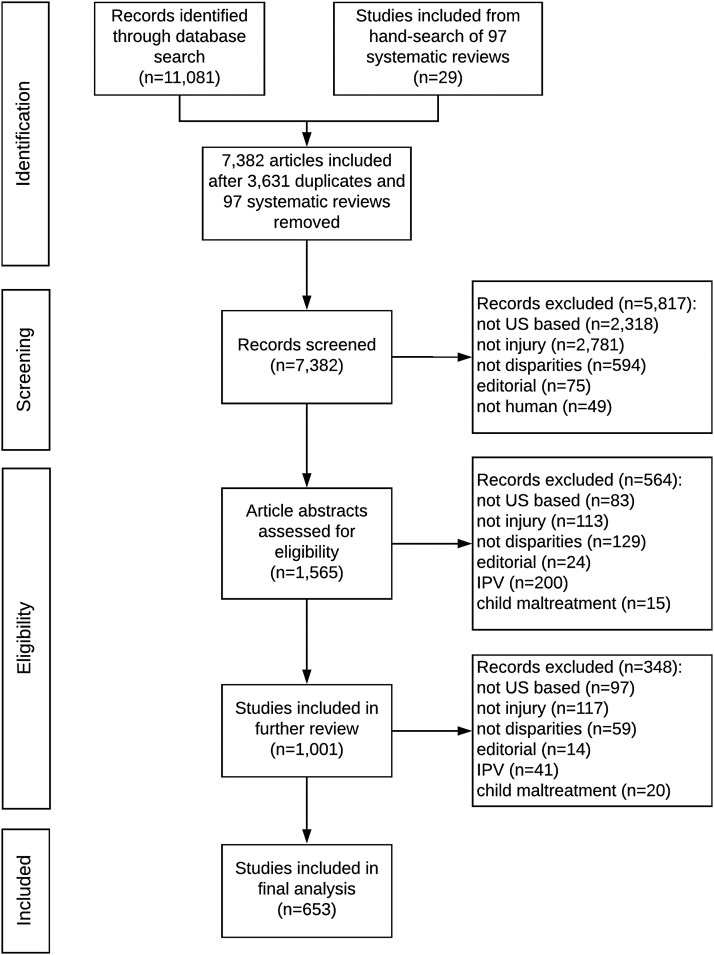
PRISMA diagram. IPV, intimate partner violence; US, United States.

Of 7382 unique articles identified in the initial search, 653 articles met inclusion criteria; 29 articles were identified using hand search of systematic reviews (Fig. 1).

### Characteristics of included studies

The number of articles published each year in the area of injury disparities nearly doubled between 2007 and 2017, from 40 articles in 2007 to 85 articles in 2015 and 78 articles in 2017. Included articles were published in 261 journals, with 2017 impact factors ranging from 0.14 to 47.7. Twelve articles were published in journals without an impact factor. The median impact factor was 1.87 (interquartile range 1.75). The journals that published 10 or more of the *N*=653 included studies were *American Journal of Public Health* (*N*=24, 3.7%), *American Journal of Industrial Medicine* (*N*=23, 3.5%), *Archives of Physical Medicine and Rehabilitation* (*N*=15, 2.3%), *Journal of Trauma* (*N*=14, 2.1%) and its new title *Journal of Trauma and Acute Care Surgery* (*N*=14, 2.1%), *Journal of Surgical Research* (*N*=13, 2.0%), *Pediatrics* (*N*=12, 1.8%), *Journal of Pediatric Surgery* (*N*=11, 1.7%), and *American Journal of Surgery* (*N*=10, 1.5%). Supplementary Table S1 provides a list of all included articles.

#### Study setting, age of study populations, and study designs

**FIG. 2.**
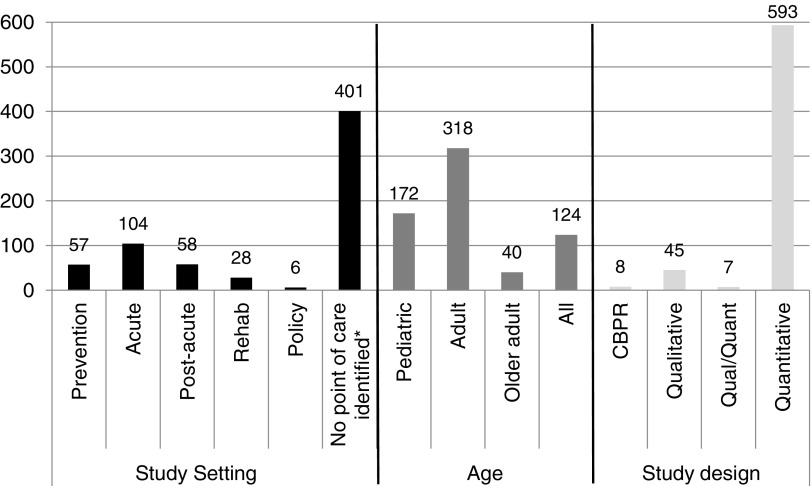
Articles by study setting, age of study populations, and study design. *These studies primarily focused on disparities in the incidence/prevalence of an injury (e.g., racial/ethnic disparities in the incidence of traumatic brain injury). CBPR, community-based participatory research.

Most articles did not focus on a particular setting (Fig. 2). Of those that did, the most commonly studied setting was acute care (e.g., acute treatment of injuries; *N*=104). Few studies focused on prevention (*N*=57) or policy (*N*=6). Many studies limited their population of interest to adults (*N*=318). The overwhelming majority of studies were quantitative (*N*=593), with 569 studies being either descriptive or comparisons between groups, 3 studies using meta-analysis, 1 study using a Delphi process, and 20 intervention studies. Of the 45 qualitative studies, 33 were conceptual studies and 12 used qualitative content analysis. Seven studies used both qualitative and quantitative analytic methods; five of those studies were intervention studies. Few studies (*N*=8) used CBPR.

#### Injury type

Using the modified injury classification developed by Cummings et al.,^20^ disparities in traumatic brain injury (TBI) were the most commonly studied, accounting for 10.6% of all articles (Table 1). Transportation injuries, falls, and firearm injuries were the most commonly studied etiological mechanisms and events. Occupational injuries were the only activity-related injury studied. Most studies that focused on intentional injuries aggregated all types of violence together, as did studies focused on injury severity.

**Table 1.** Classification of Included Studies

	No. of articles (*N*)	Percent (%)
Anatomy/pathology		
Traumatic brain injury	69	10.6
Musculoskeletal injury	34	5.2
Spinal injury^a^	31	4.7
Burn	6	0.9
Abdominal/pelvic injury	4	0.6
Vascular injury	3	0.5
Laceration	2	0.3
Oral injury	1	0.2
Etiologic mechanism or event		
Transportation^b^	43	6.6
Fall	26	4.0
Firearm	18	2.8
Drowning	4	0.6
Dog bite	2	0.3
Poisoning	1	0.2
Weather	1	0.2
Location/activity where injury occurred		
Workplace/occupation	70	10.7
Intent		
Aggregate violence^c^	105	16.1
Homicide	30	4.6
Suicide/self-directed	28	4.3
School violence^d^	15	2.3
Sexual violence	14	2.1
Legal intervention homicide/injuries	11	1.7
Violent hate crimes^e^	3	0.5
Severity		
Studies in which injury (aggregate) severity was a primary focus^f^	98	15.0
Other		
Aggregrate injury (e.g., study of trauma care providers' biases)	31	4.7

^a^ Includes spinal cord injury and vertebral injury.

^b^ Transportation injuries include safety restraints (e.g., booster seats and seat belts; *N*=14, 2.1%), helmets (*N*=4, 0.6%), motor vehicle crash (*N*=22, 3.4%), and other transportation injury (*N*=3, 0.5%).

^c^ Includes studies that either did not disaggregate intent or mechanism of violence or reported on multiple intents or mechanisms of violence.

^d^ Includes all intents and mechanisms of violence that occur at school, including bullying, gang violence, and unspecified violent crimes at school.

^e^ To be included, studies must either only report on violent hate crimes (e.g., assault) or disaggregate property crimes from physical assault.

^f^ These studies did not disaggregate by anatomical location/pathology, etiological mechanism, event, location/activity, or intent and defined injury by severity (e.g., Injury Severity Score).

#### Disparity type

**FIG. 3.**
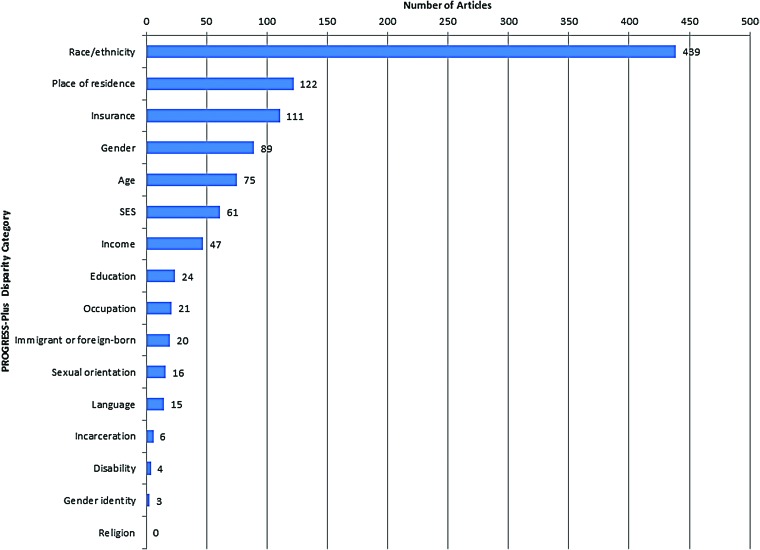
Number of articles reporting on each PROGRESS-Plus disparity category. Categories not mutually exclusive. SES, socioeconomic status.

Racial disparities were the most commonly examined disparities (*N*=439, 67.2%; Fig. 3). Of these, most studies considered disparities for African American and/or Hispanic persons either compared with white persons or others or population averages (*N*=306, 46.8%). Of note, four studies (0.9%) delineated Asian ethnicities, and 11 (2.5%) studies delineated Native Hawaiian or Pacific Islander from the Asian race. Thirty-eight (8.7%) studies created white/nonwhite binary racial categories.

Place of residence was also a commonly studied disparity (122, 18.7%). These articles most commonly considered disparities in injury among different neighborhoods (42, 6.4%) or by rurality (28, 4.3%). Other studies considered disparities based on insurance (111, 17.0), gender (89, 13.6%), or age (75, 11.5%); some studies further defined SES by income, education, and other factors. Few studies reported on disparities by sexual orientation or gender identity, language, incarceration status, housing, disability, or religion.

## Discussion

Growing attention to disparities in injury is evidenced by a nearly twofold increase in research studies published each year over the last decade. Research to date has largely focused on disparities in the acute care setting, an important area that is now ready for intervention development to address disparities. However, preventing injuries in high-risk groups could have the biggest influence on achieving health equity in injury. Few studies have explicitly explored strategies to promote health equity. Some examples of public health injury prevention campaigns to address disparities include a booster seat campaign developed by and tailored to Latino children^21,22^ and an effort to provide window locks to families with children at high risk for window falls, a campaign that resulted from an observational study.^23^ More research that is followed by public health prevention campaigns to address the disparity identified is urgently needed to fill these gaps.

In addition, postacute care and rehabilitation are critically important areas for research on disparities, yet these areas are understudied. Disparities in access to and use of rehabilitation services have been documented;^24,25^ identifying effective strategies to better link vulnerable patients to postacute care, using policy changes or hospital- and community-level interventions, is a promising avenue for further exploration. For example, one study recommended universal inpatient assessment of rehabilitation needs to decrease disparities in outpatient rehabilitation access for children with TBI.^26^ Finally, few studies have focused on policy development to address disparities. Policy development has the potential to decrease injuries and injury disparities, as evidenced by research on primary enforcement laws for safety belt and booster seat use.^27–29^ Policy development should consider both the differential positive impact and challenges for vulnerable groups. For example, policies that aim to increase booster seat use may not consider that income disparities reduce access to booster seats^29^ or some groups have less access to knowledge about traffic laws,^30^ which may put them at risk for both injury and unequal enforcement of the law.

Published research has identified injury-related disparities among many different age groups. Most studies identified in this review focused on adult patients, with fewer studies on children and adolescents, and even fewer on older adults. Children, adolescents, and older adults are at high risk for injury and have unique needs related to recovery; additional research to address the needs of these groups is important.

Conducting impactful research on disparities in injury requires availability of comprehensive, specific, accurate, and inclusive data. Data sources with indicators for diverse patient groups as well as comprehensive injury and outcome data are limited. For instance, while many studies assessed disparities by racial/ethnic group, most of these studies relied upon racial or ethnic categories from administrative data, which may not be accurately reported and significantly limit the specificity of racial groups and documentation of persons who identify as more than one race. While findings from this research indicate that racial and ethnic disparities in injury exist across the care spectrum, from risk of injury to postacute care access and outcomes, we found that there were additional limitations in data categories. Specifically, many studies aggregated racial/ethnic groups. Further disaggregating these groups will assist in better understanding the unique needs of patients and their families. Using meaningful categories of race and ethnicity, language, and other equity-related measures in our trauma databases at the local and national levels can assist in development of culturally responsive interventions. In addition, many studies identified in this review also aggregated injury types and injury intent (e.g., types of violence), limiting our ability to specify potential actions for alleviating disparities. Interventions to address these disparities and interventions that are developed using a community participatory strategy are key to achieving health equity in injury.^6^

Research to date has also explored disparities by place of residence, demonstrating higher risk and poorer outcomes for rural residents and those living in high-poverty areas.^31,32^ Exploring strategies to address these disparities are in their infancy. In some research and service areas, focused systematic reviews would be helpful in determining gaps and areas of focus. For instance, a review of the literature in occupational injury disparities would be helpful. Several important categories of difference remain understudied, including disparities by sexual and gender identities, language, disability status, and religion, among others. Understanding the differential effect of injury on diverse groups of people is a key step toward improving care and enhancing services to groups most in need.

To conduct research that adequately addresses the needs of vulnerable groups, researchers must diversify the methods used to understand and address disparities. To date, few studies have focused on prevention, policy or intervention development, and testing. Expanding future research to include community-based participatory strategies and intervention research are urgently needed to enhance our current knowledge and expand the impact of injury research. In addition, policy makers, researchers, and providers can enhance engagement with community stakeholders through development of community advisory boards and other community-based participatory strategies.^33,34^ These strategies have been shown to improve engagement and the reach of research and practice strategies aimed at improving care for marginalized groups.^33,34^

### Limitations

It is possible that relevant studies were not included despite our use of expert librarians to assist with search terms and our close following of the PRISMA Extension for Scoping Reviews. Our research questions were exploratory in nature, and we were interested in studies that clearly defined a physical injury and disparity, as defined by the Cochrane PROGRESS-Plus criteria, limiting the scope of the review. Because of the large number of studies, we could not perform an assessment of study quality beyond the journal impact factor proxy.

## Conclusions

There are disparities in injury across the care spectrum and all categories of the PROGRESS-Plus-defined disparities studied. More research is urgently needed in many categories of difference. Coordinating efforts across the field to collect and report on meaningful health equity variables homogeneously will assist in increasing comparability and quality of studies. Improving the quality, specificity, and availability of data relevant to decreasing disparities is of paramount importance. The largely descriptive research to date provides the first and necessary step toward understanding disparities. Policy development, tailored public health prevention campaigns, and intervention research using community participatory approaches are promising next steps in the progression of the field to achieve injury-related health equity and reduction of injury disparities.

## Supplementary Material

Supplemental data

Supplemental data

## Abbreviations Used

CBPR: community-based participatory research
SES: socioeconomic status
TBI: traumatic brain injury
IPV: intimate partner violence
