# Supplementary material for: Research on Injury Disparities: A Scoping Review
Source: Health Equity. 2019 Oct 17;3(1):504–11. doi: 10.1089/heq.2019.0044 (PMC6798805; doi:10.1089/heq.2019.0044)
Supplement: Supplemental data [file Suppl_Data.pdf]

Supplementary Data

Supplementary Data: Databases, Search Terms, and Parameters

PubMed search

1. ("Healthcare Disparities" [Mesh] OR health status disparities [mesh] OR disparity [tiab] OR disparities [tiab] OR equit* [tiab] OR inequit* [tiab] OR inequalit* [tiab]) AND (injury [tiab] OR injuries [tiab] OR trauma* [tiab] OR violence [tiab] OR wounds and injuries [mesh])

   - Parameters
     - 2007–2010
     - Human subjects

2. (("Healthcare Disparities" [Mesh] OR health status disparities [mesh] OR disparity[tiab] OR disparities [tiab] OR equit* [tiab] OR inequit* [tiab] OR inequalit* [tiab]) AND (injury [tiab] OR injuries [tiab] OR trauma* [tiab] OR violence [tiab] OR wounds and injuries [mesh]) AND 2007:2017 [pdat] NOT (animals [mh] NOT humans [mh])) NOT (("Healthcare Disparities" [Mesh] OR health status disparities [mesh] OR disparity [tiab] OR disparities[tiab] OR equit*[tiab] OR inequit*[tiab] OR inequalit*[tiab]) AND (injury [tiab] OR injuries [tiab] OR trauma* [tiab] OR violence [tiab] OR wounds and injuries [mesh]) AND 2007:2017 [pdat] AND humans [mh])

CINAHL

3. (MH "Healthcare Disparities" OR MH "Health Status Disparities" OR disparity OR disparities OR equit* OR inequit* OR inequalit*) AND (injury OR injuries OR trauma* OR violence OR MH "Violence" OR MH "Wounds and Injuries + ") AND MX Y

Web of Science

4. (disparity or equity or inequity or inequality) AND (health OR healthcare) AND (injury or trauma or violence)

PsycINFO

5. (MH "Healthcare Disparities" OR MH "Health Status Disparities" OR disparity OR disparities OR equit* OR inequit* OR inequalit*) AND (injury OR injuries OR trauma* OR violence OR MH "Violence" OR MH "Wounds and Injuries + ")
