# Supplementary material for: Research on Injury Disparities: A Scoping Review
Source: Health Equity. 2019 Oct 17;3(1):504–11. doi: 10.1089/heq.2019.0044 (PMC6798805; doi:10.1089/heq.2019.0044)
Supplement: Supplemental data [file Suppl_TableS1.docx]

| **Supplemental Table 1. Included Articles** | | | |
| --- | --- | --- | --- |
| **First Author** | **Title** | **Year** | **Journal** |
| Adanu EK | Multilevel analysis of the role of human factors in regional disparities in crash outcomes | 2017 | Accident Analysis and Prevention |
| Ahmed | Not Just an Urban Phenomenon: Uninsured Rural Trauma Patients at Increased Risk for Mortality | 2015 | The Western Journal of Emergency Medicine |
| ALamgir | Unintentional falls mortality among elderly in the United States: time for action | 2012 | Injury |
| Alban | Does Health Care Insurance Affect Outcomes after Traumatic Brain Injury? Analysis of the National Trauma Databank | 2010 | The American Journal of Surgery |
| Albrecht | Patterns of depression treatment in medicare beneficiaries with depression after traumatic brain injury | 2015 | Journal of Neurotrauma |
| Alghnam | Insurance status and health-related quality-of-life disparities after trauma: Results from a nationally representative survey in the US | 2016 | Quality of Life Research |
| Ali | Socioeconomic disparity in inpatient mortality after traumatic injury in adults | 2013 | Surgery |
| Allen et al | Playground safety and quality in Chicago | 2013 | Pediatrics |
| Alvidrez | Which low-income urban crime victims use trauma-focused case management and psychotherapy services? | 2008 | Journal of Loss and Trauma |
| Alvidrez | Ethnic disparities in mental health treatment engagement among female sexual assault victims | 2011 | Journal of Aggression, Maltreatment, and Trauma |
| Alvidrez | Reduction of state victim compensation disparities in disadvantaged crime victims through active outreach and assistance: a randomized trial | 2008 | American Journal of Public Health |
| Amanullah | Differences in Presentation and Management of Pediatric Facial Lacerations by Type of Health Insurance | 2015 | The Western Journal of Emergency Medicine |
| Anderson KL | Traffic law knowledge disparity between Hispanics and non-Hispanic whites in California | 2011 | Journal of Emergency Medicine |
| Apostolico | Injury surveillance and associations with socioeconomic status indicators among youth/young workers in New Jersey secondary schools | 2016 | Environmental Health |
| Arango-Lasprilla | Functional outcomes from inpatient rehabilitation after traumatic brain injury: How do Hispanics fare? | 2007 | Archives of Physical Medicine and Rehabilitation |
| Arango-Lasprilla | Influence of minority status on job stability after traumatic brain injury | 2009 | Physical Medicine and Rehabilitation |
| Arango-Lasprilla | Racial differences in employment outcomes after traumatic brain injury. | 2008 | Archives of Physical Medicine and Rehabilitation |
| Arango-Lasprilla | Traumatic brain injury and functional outcomes: Does minority status matter? | 2007 | Brain Injury |
| Arango-Lisprilla | Ethnicity/racial differences in employment outcomes following spinal cord injury | 2009 | NeuroRehabilitation |
| Arango-Lisprilla | Racial and ethnic disparities in employment outcomes for persons with traumatic brain injury: a longitudinal investigation 1-5 years after injury | 2011 | Physical Medicine and Rehabilitation |
| Archambeau | Interpersonal violence and mental health outcomes among Asian American and Native Hawaiian/other Pacific Islander college students | 2010 | Psychological Trauma: Theory, Research, Practice, and Policy |
| Arcury | Personal protective equipment and work safety climate among Latino poultry processing workers in Western North Carolina, USA | 2012 | International Journal of Occupational and Environmental Health |
| Arcury | Employer, use of personal protective equipment, and work safety climate: Latino poultry processing workers | 2013 | American Journal of Industrial Medicine |
| Arcury | Musculoskeletal and neurological injuries associated with work organization among immigrant Latino women manual workers in North Carolina | 2014 | American Journal of Industrial Medicine |
| Arcury | Work safety climate, musculoskeletal discomfort, working while injured, and depression among migrant farmworkers in North Carolina | 2012 | American Journal of Public Health |
| Arias | Disparities in Treatment of Older Adults with Suicide Risk in the Emergency Department | 2017 | Journal of the American Geriatrics Society |
| Arthur et al | Racial disparities in mortality among adult hospitalized after injury | 2008 | Medical Care |
| Asemota et al | Race and insurance disparities in discharge to rehabilitation for patients with traumatic brain injury | 2013 | Journal of Neurotrauma |
| Azur | Violent and nonviolent delinquent behavior among Caucasian and Hispanic youth in mental health systems-of-care programs | 2011 | Youth Violence and Juvenile Justice |
| Bachier-Rodriquez | Firearm injuries in a pediatric population: African-American adolescents continue to carry the heavy burden | 2017 | The American Journal of Surgery |
| Baker et al | A source of healthcare disparity: race, skin color, and injuries after rape among adolescents and young adults | 2010 | Journal of Forensic Nursing |
| Barlow | Understanding the relationship between substance use and self-injury in American Indian youth | 2012 | The American Journal of Drug and Alcohol Abuse |
| Barrett | Exploring Community Levels of Lead (Pb) and Youth Violence | 2017 | Sociological Spectrum |
| Bauerle | Mere overrepresentation? Using cross-occupational injury and job analysis data to explain men’s risk for workplace fatalities | 2016 | Safety Science |
| Bayouth L | An in-situ simulation-based educational outreach project for pediatric trauma care in a rural trauma system | 2017 | Journal of Pediatric Surgery |
| Bazargan-Hejazi S | Profile of Hospital Admissions due to Self-Inflicted Harm in Los Angeles County from 2001 to 2010 | 2017 | Journal of Forensic Science |
| Beard | Quantifying disparities in urban firearm violence by race and place in Philadelphia, Pennsylvania: A cartographic study | 2017 | American Journal of Public Health |
| Beardslee J | Childhood risk factors associated with adolescent gun carrying among Black and White males: An examination of self-protection, social influence, and antisocial propensity explanations | 2017 | Law and Human Behavior |
| Beck | Areas with High Rates of Police-Reported Violent Crime Have Higher Rates of Childhood Asthma Morbidity | 2016 | The Journal of Pediatrics |
| Beck | Rural and Urban Differences in Passenger-Vehicle-Occupant Deaths and Seat Belt Use Among Adults - United States, 2014 | 2017 | MMWR Morbidity and Mortality Weekly Report |
| Beck | Associations between sociodemographics and safety belt use in states with and without primary enforcement laws | 2007 | American Journal of Public Health |
| Bedri | A National Study of the Effect of Race, Socioeconomic Status, and Gender on Burn Outcomes | 2017 | Journal of Burn Care & Research |
| Bekelis | The Association of Insurance Status and Race With Transfers of Patients With Traumatic Brain Injury Initially Evaluated at Level III and IV Trauma Centers | 2015 | Annals of Surgery |
| Bell et al | Insurance status is a predictor of failure to rescue in trauma patients at both safety net and non-safety net hospitals | 2013 | Journal of Trauma and Acute Care Surgery |
| Bell et al | Disparities in disability after traumatic brain injury causes in the USA: a review | 2015 | Pediatrics |
| Bell et al | Census-based socioeconomic indicators for monitoring injury causes in the USA: a review | 2015 | Injury Prevention |
| Bell N | The reliability of the American Community Survey for injury surveillance | 2017 | Injury Prevention |
| Berdahl | Occupational racial composition and nonfatal work injuries | 2008 | Social Problems |
| Berdahl | Racial/ethnic and gender differences in individual workplace injury risk trajectories: 1988-1998 | 2008 | American Journal of Public Health |
| Berdahl | Medical care utilization for work-related injuries in the United States 2002–2006 | 2010 | Medical Care |
| Berg | Accounting for Racial Disparities in the Nature of Violent Victimization | 2014 | Journal of Quantitative Criminology |
| Berg et al | The effect of methodology in determining disparities in in-hospital mortality of trauma patients based on payer source | 2015 | Journal of Trauma Nursing |
| Bernard SJ | Fatal injuries among children by race and ethnicity--United States, 1999-2002 | 2007 | MMWR Morbidity and Mortality Weekly Report |
| Berns | Development of a culturally informed child safety curriculum for American Indian families | 2017 | The Journal of Primary Prevention |
| Berry et al | Race affects mortality after moderate to severe traumatic brain injury | 2010 | Journal of Surgical Research |
| Berthelot | Racial (In)variance, Disadvantage, and Lethal Violence: A Survival Analysis of Black Homicide Victimization Risk in the United States | 2016 | Homicide Studies |
| Bijur | Race, ethnicity, and management of pain from long-bone fractures: a prospective study of two academic urban emergency departments | 2008 | Academic Emergency Medicine |
| Bijur | No racial or ethnic disparity in treatment of long-bone fractures | 2008 | American Journal of Emergency Medicine |
| Billie | Child safety and booster seat use in five tribal communities, 2010–2014 | 2016 | Journal of Safety Research |
| Bishai | Heightened risk of fire deaths among older African Americans and Native Americans | 2010 | Public Health Reports |
| Bjork | Dog bite injuries among American Indian and Alaska Native children | 2013 | The Journal of Pediatrics |
| Bjurlin | Race and insurance status are risk factors for orchiectomy due to testicular trauma | 2012 | Journal of Urology |
| Blosnich | Associations of discrimination and violence with smoking among emerging adults: Differences by gender and sexual orientation | 2011 | Nicotine and Tabacco Research |
| Blosnich J | Drivers of disparity: differences in socially based risk factors of self-injurious and suicidal behaviors among sexual minority college students | 2012 | Journal of American College Health |
| Bolorunduro et al | Disparities in trauma care: are fewer diagnostic tests conducted for uninsured patients with pelvic fracture? | 2013 | American Journal of Surgery |
| Bonauto | Language Preference and Non-Traumatic Low Back Disorders in Washington State Workers' Compensation | 2010 | American Journal of Industrial Medicine |
| Bonugli | "The second thing to hell is living under that bridge": narratives of women living with victimization, serious mental illness, and in homelessness | 2013 | Issues in Mental Health Nursing |
| Bose | Vulnerability of female drivers involved in motor vehicle crashes: An analysis of US population at risk | 2011 | American Journal of Public Health |
| Botticello | To What Extent Do Neighborhood Differences Mediate Racial Disparities in Participation After Spinal Cord Injury? | 2016 | Archives of Physical Medicine and Rehabilitation |
| Bouris | Effects of Victimization and Violence on Suicidal Ideation and Behaviors Among Sexual Minority and Heterosexual Adolescents | 2016 | Lgbt Health |
| Bowman SM | Racial disparities in outcomes of persons with moderate to severe traumatic brain injury | 2007 | Medical Care |
| Boyer | Ergonomic and Socioeconomic Risk Factors for Hospital Workers' Compensation Injury Claims | 2009 | American Journal of Industrial Medicine |
| Boykins | Minority Women Victims of Recent Sexual Violence: Disparities in Incident History | 2010 | Journal of Women's Health |
| Boynton-Jarrett | Cumulative violence exposure and self-rated health: longitudinal study of adolescents in the United States | 2008 | Pediatrics |
| Braithwaite | Sexual violence in the backlands: Toward a macro-level understanding of rural sex crimes | 2015 | Sexual Abuse |
| Branch | Assessment of racial and sex disparities in open femoral fractures | 2015 | American Journal of Surgery |
| Bridge | Suicide Trends Among Elementary School-Aged Children in the United States From 1993 to 2012 | 2015 | Jama Pediatrics |
| Brodzinski | Assessment of disparities in the use of anxiolysis and sedation among children undergoing laceration repair | 2010 | Academic Pediatrics |
| Brown | Geographic Variation in Outcome Benefits of Helicopter Transport for Trauma in the United States: A Retrospective Cohort Study | 2016 | Annals of Surgery |
| Brown | Geographic distribution of trauma centers and injury-related mortality in the United States | 2016 | Journal of Trauma and Acute Care Surgery |
| Brown SA | Racial Disparities in Depression and Life Satisfaction After Spinal Cord Injury: A Mediational Model | 2012 | Top Spinal Cord Inj Rehabil |
| Browning CR | Understanding Racial Differences in Exposure to Violent Areas: Integrating Survey, Smartphone, and Administrative Data Resources | 2017 | Annals of the American Academy of Political and Social Science |
| Buchanan | Occupational injury disparities in the US hotel industry | 2010 | American Journal of Industrial Medicine |
| Buehler | Racial/ethnic disparities in the use of lethal force by US police, 2010–2014 | 2017 | American Journal of Public Health |
| Carbado | From Stop and Frisk to Shoot and Kill: Terry v Ohio's Pathway to Police Violence | 2017 | UCLA Law Review |
| Carlos Arango-Lasprilla | Vocational Rehabilitation Service Patterns and Employment Outcomes for Hispanics with Spinal Cord Injuries | 2011 | Rehabilitation Research, Policy, and Education |
| Carlyle | Demographic differences in the prevalence, co‐occurrence, and correlates of adolescent bullying at school | 2007 | Journal of School Health |
| Carter | Firearm violence among high-risk emergency department youth after an assault injury | 2015 | Pediatrics |
| Cary et al | Inpatient rehabilitation outcomes in a national sample of Medicare beneficiaries with hip fracture | 2016 | Journal of Applied Gerontology |
| Cassidy et al | Health disparities analysis of critically ill pediatric trauma patients in Milwaukee, Wisconsin | 2013 | Journal of the American College of Surgeons |
| Castrodale | Hospitalizations resulting from dog bite injuries - Alaska, 1991-2002 | 2007 | International Journal of Circumpolar Health |
| CDC | Alcohol and suicide among racial/ethnic populations—17 states, 2005–2006 | 2009 | MMWR Morbidity and Mortality Weekly Report |
| Chaffin | Same-sex and race-based disparities in statutory rape arrests | 2016 | Journal of Interpersonal Violence |
| Chan | Pediatric facial fractures: Demographic determinants influencing clinical outcomes | 2016 | The Laryngoscope |
| Chan V | Clinical profile and comorbidity of traumatic brain injury among younger and older men and women: a brief research notes | 2017 | BMC Research Notes |
| Chang | Ethnic differences in discharge destination among older patients with traumatic brain injury. | 2008 | Archives of Physical Medicine and Rehabilitation |
| Chapleau | A system justification view of sexual violence: Legitimizing gender inequality and reduced moral outrage are connected to greater rape myth acceptance | 2014 | Journal of Trauma & Dissociation |
| Chaudhary MA | Universal insurance and an equal access healthcare system eliminate disparities for Black patients after traumatic injury | 2017 | Surgery |
| Cherpitel | Relationship of usual volume and heavy consumption to risk of alcohol-related injury: racial/ethnic disparities in four US national alcohol surveys | 2016 | Journal of Studies on Alcohol and Drugs |
| Chikani | Racial/Ethnic Disparities in Rates of Traumatic injury in Arizona, 2011-2012 | 2016 | Public Health Reports |
| Chikani et al | Association of insurance status with health outcomes following traumatic injury: statewide multicenter analysis | 2015 | The Western Journal of Emergency Medicine |
| Choi | Ethnic disparities in end-of-life care after subarachnoid hemorrhage | 2015 | Neurocritical Care |
| Chong | Neighborhood socioeconomic status is associated with violent reinjury | 2015 | Journal of Surgical Research |
| Chong et al | Potential disparities in trauma: the undocumented Latino immigrant | 2014 | Journal of Surgical Research |
| Chung | The relationship between ASSH membership and the treatment of distal readius fracture in the United States Medicare population | 2011 | Journal of Hand Surgery |
| Chung-Do | Understanding the role of school connectedness and its association with violent attitudes and behaviors among an ethnically diverse sample of youth | 2017 | Journal of Interpersonal Violence |
| Cigularov | Measurement equivalence of a safety climate measure among Hispanic and White Non-Hispanic construction workers | 2013 | Safety Science |
| Clark | Vocational interests by gender and race 10 years after spinal cord injury | 2017 | Rehabilitation Psychology |
| Clark et al | Estimating the effect of emergency care on early survival after traffic crashes | 2013 | Accident Analysis and Prevention |
| Cohen L | Communities are not all created equal: Strategies to prevent violence affecting youth in the United States | 2016 | Journal of Public Health Policy |
| Cook A | No Disparity for American Indians in Surgery for Pelvis/Lower Extremity Fractures: a Cohort Study of the National Trauma Data Bank (NTDB) | 2017 | Journal of Racial and Ethnic Health Disparities |
| Cook AD | Race and rehabilitation following spinal cord injury: equality of access for American Indians/Alaska Natives compared to other racial groups | 2015 | Injury Epidemiology |
| Cooper et al | Racial disparities in intensity of care at the end-of-life: are trauma patients the same as the rest? | 2012 | Journal of Health Care for the Poor and Underserved |
| Costanza | Street Gangs and Aggregate Homicides: An Analysis of Effects During the 1990s Violent Crime Peak | 2012 | Homicide Studies |
| Crandall et al | Lower extremity vascular injuries: increased mortality for minorities and the uninsured? | 2011 | Surgery |
| Crisanti | Ethnoracial disparities in sexual assault among Asian Americans and Native Hawaiians/other Pacific Islanders | 2011 | The Journal of Clinical Psychiatry |
| Crompton et al | Racial disparities in motorcycle-related mortality: an analysis of the National Trauma Data Bank | 2010 | American Journal of Surgery |
| Crosslin | Acculturation in Hispanics and childhood poisoning: Are medicines and household cleaners stored properly? | 2011 | Accident Analysis and Prevention |
| Crutcher | Racial Disparities in Cranial Gunshot Wounds: Intent and Survival | 2016 | Journal of Racial and Ethnic Health Disparities |
| Curry | Variation in teen driver education by state requirements and sociodemographics | 2012 | Pediatrics |
| Curtis | Population-based fracture risk assessment and osteoporosis treatment disparities by race and gender | 2009 | Journal of General Internal Medicine |
| Cuthbert | Factors That Predict Acute Hospitalization Discharge Disposition for Adults With Moderate to Severe Traumatic Brain Injury | 2011 | Archives of Physical Medicine and Rehabilitation |
| da Silva Cardoso E | Disparities in vocational rehabilitation services and outcomes for Hispanic clients with traumatic brain injury: do they exist? | 2007 | Journal of Head Trauma Rehabilitation |
| Dai | Childhood drowning in Georgia: A geographic information system analysis | 2013 | Applied Geography |
| D'Alessio | Racial animosity and interracial crime | 2009 | Criminology: An Interdisciplinary Journal |
| Daly | The Influence of Insurance Status on the Surgical Treatment of Acute Spinal Fractures | 2016 | Spine |
| Davis | Treatment barriers for low-income, urban African Americans with undiagnosed posttraumatic stress disorder | 2008 | Journal of Traumatic Stress |
| de la Plata | Ethnic differences in rehabilitation placement and outcome after TBI | 2007 | Journal of Head Trauma Rehabilitation |
| Decker | Sexual violence against adolescent girls - Influences of immigration and acculturation | 2007 | Violence Against Women |
| DeCou CR | The benefits of discussing suicide with Alaska native college students: qualitative analysis of in-depth interviews | 2013 | Cultural Diversity and Ethnic Minority Psychology |
| Delgado | Factors Associated With the Disposition of Severely Injured Patients Initially Seen at Non-Trauma Center Emergency Departments Disparities by Insurance Status | 2014 | Jama Surgery |
| Denney | The social side of accidental death | 2014 | Social Science Research |
| DeRigne L | Workers Without Paid Sick Leave Less Likely To Take Time Off For Illness Or Injury Compared To Those With Paid Sick Leave | 2016 | Health Affairs |
| Desai | Mental illness, violence risk, and race in juvenile detention: Implications for disproportionate minority contact | 2012 | American Journal of Orthopsychiatry |
| DeVylder JE | Prevalence, demographic variation and psychological correlates of exposure to police victimisation in four US cities | 2017 | Epidemiology and Psychiatric Sciences |
| Dicker | Where do we go from here? Interim analysis to forge ahead in violence prevention | 2009 | Journal of Trauma |
| Diem | Social Structure and Family Homicides | 2010 | Journal of Family Violence |
| Dillahunt-Aspillaga | Predictors of behavioural health service use and associated expenditures: Individuals with TBI in Pinellas County | 2015 | Brain Injury |
| Dismuke | Racial/Ethnic Differences in Combat- and Non-Combat-Associated Traumatic Brain Injury Severity in the Veterans Health Administration: 2004-2010 | 2015 | American Journal of Public Health |
| Dismuke | Racial/Ethnic Disparities in VA Services Utilization as a Partial Pathway to Mortality Differentials Among Veterans Diagnosed With TBI | 2015 | Global Journal of Health Science |
| Doctor | Socioeconomic Status and Outcomes After Burn Injury | 2016 | Journal of Burn Care & Research |
| Dominguez | Beyond individual and visible acts of violence: A framework to examine the lives of women in low-income neighborhoods | 2014 | Women's Studies International Forum |
| Dong | Medical costs and sources of payment for work-related injuries among Hispanic construction workers | 2007 | Journal of Occupational and Environmental Medicine |
| Dong et al | Work-related injuries among Hispanic construction workers-evidence from the medical expenditure panel survey | 2010 | American Journal of Industrial Medicine |
| Dong XS | Fatal falls among Hispanic construction workers | 2009 | Accident Analysis and Prevention |
| Donner WR | The political ecology of disaster: an analysis of factors influencing US tornado fatalities and injuries, 1998-2000 | 2007 | Demography |
| Dowell | Socioeconomic Factors Are Associated With Trends in Treatment of Pediatric Femoral Shaft Fractures, and Subsequent Implant Removal in New York State | 2016 | Journal of Pediatric Orthopaedics |
| Downing | The impact of insurance status on actuarial survival in hospitalized trauma patients: when do they die? | 2011 | Journal of Trauma |
| Dozier | Insurance Coverage Is Associated with Mortality after Gunshot Trauma | 2010 | Journal of the American College of Surgeons |
| Driesman A | Race and Ethnicity Have a Mixed Effect on the Treatment of Tibial Plateau Fractures | 2017 | Journal of Orthopedic Trauma |
| Du | Incidence of workers compensation indemnity claims across socio-demographic and job characterisitcs | 2011 | American Journal of Industrial Medicine |
| Duncan | Lesbian, gay, bisexual, and transgender hate crimes and suicidality among a population-based sample of sexual-minority adolescents in Boston | 2014 | American Journal of Public Health |
| Dunn | The impact of exposure to interpersonal violence on gender differences in adolescent‐onset major depression: Results from the National Comorbidity Survey Replication (NCS-R) | 2012 | Depression and Anxiety |
| duron et al | undiagnosed medical comorbidities in the uninsured: a significant predictor of mortality following trauma | 2012 | Journal of Trauma and Acute Care Surgery |
| Dy | Racial and Socioeconomic Disparities in Hip Fracture Care | 2016 | Journal of Bone and Joint Surgery |
| Dy | Socioeconomic factors are associated with frequency of repeat emergency department visits for pediatric closed fractures | 2014 | Journal of Pediatric Orthopaedics |
| Eaton | Associations Between Risk Behaviors and Suicidal Ideation and Suicide Attempts: Do Racial/Ethnic Variations in Associations Account for Increased Risk of Suicidal Behaviors Among Hispanic/Latina 9th- to 12th-Grade Female Students? | 2011 | Archives of Suicide Research |
| Echeverria | A community survey on neighborhood violence, park use, and physical activity among urban youth | 2014 | Journal of Physical Activity & Health |
| Edberg | SAFER Latinos: a community partnership to address contributing factors for Latino youth violence | 2010 | Progress in Community Health Partnerships: Research, Education, and Action |
| Egede | Racial/ethnic disparities in mortality risk among US veterans with traumatic brain injury | 2012 | American Journal of Public Health |
| Ehrsam | Alaska Native Mortality Rates and Trends | 2009 | Public Health Reports |
| Eisman AB | Sexual Violence Victimization Among Youth Presenting to an Urban Emergency Department: The Role of Violence Exposure in Predicting Risk | 2017 | Health Education & Behavior |
| Eitle | Public school segregation and juvenile violent crime arrests in metropolitan areas | 2010 | Sociological Quarterly |
| Embrick | Two Nations, Revisited: The Lynching of Black and Brown Bodies, Police Brutality, and Racial Control in 'Post-Racial' Amerikkka | 2015 | Critical Sociology |
| Englum | Association Between Insurance Status and Hospital Length of Stay Following Trauma | 2016 | American Surgeon |
| Englum et al | Racial, ethnic, and insurance status disparities in use of posthospitalization care after trauma | 2011 | Journal of the American College of Surgeons |
| Engrav | Harborview Burns-1974 to 2009 | 2012 | Plos One |
| Ertl A | Characteristics of Traumatically Injured Pediatric Assault Patients: A Statewide Assesment in Ohio | 2017 | Journal of Registry Management |
| Estell | Bullies and victims in rural African American youth: Behavioral characteristics and social network placement | 2007 | Aggressive Behavior |
| Estrada-Martínez | Adolescent neighborhood environments and Latino intraethnic disparities in trajectories of serious violent behaviors | 2017 | Hispanic Journal of Behavioral Sciences |
| Fagenholz | National study of emergency department visits for burn injuries, 1993 to 2004 | 2007 | Journal of Burn Care & Research |
| Falcone | Despite overall low pediatric head injury mortality, disparitiesexist between races | 2008 | Journal of Pediatric Surgery |
| Falcone RA Jr | The epidemiology of infant injuries and alarming health disparities | 2007 | Journal of Pediatric Surgery |
| Fanuele | Variation in hip fracture treatment: are black and white patients treated equally? | 2009 | American Journal of Orthopsychiatry |
| Fanuele | Distal radial fracture treatment: what you get may depend on your age and address | 2009 | Journal of Bone and Joint Surgery |
| Farrell | Symptoms and Time to Medical Care in Children With Accidental Extremity Fractures | 2012 | Pediatrics |
| Feldman JM | Temporal Trends and Racial/Ethnic Inequalities for Legal Intervention Injuries Treated in Emergency Departments: US Men and Women Age 15-34, 2001-2014 | 2016 | Journal of urban health |
| Feldmeyer | Immigration Effects on Homicide Offending For Total and Race/Ethnicity-Dis aggregated Populations (White, Black, and Latino) | 2009 | Homicide Studies |
| Feldmeyer B | Racial/Ethnic Composition and Violence: Size-of-Place Variations in Percent Black and Percent Latino Effects on Violence Rates | 2013 | Sociological Forum |
| Felson | Do theories of crime or violence explain race differences in delinquency? | 2008 | Social Science Research |
| Felton et al | Unintentional, non-fatal drowning of children: US trends and racial/ethnic disparities | 2015 | BMJ Open |
| Ferrada et al | the uninsured, the homeless, and the undocumented immigrant trauma patient, revealing health-care disparitiy at a level 1 trauma center | 2016 | American Surgeon |
| Fitzpatrick | Bullying and depressive symptomatology among low‐income, African–American youth | 2010 | Journal of Youth and Adolescence |
| Fitzpatrick | Not just pushing and shoving: School bullying among African American adolescents | 2007 | Journal of School Health |
| Flottemesch | Age-related Disparities in Trauma Center Access for Severe Head Injuries Following the Release of the Updated Field Triage Guidelines | 2017 | Academic Emergency Medicine |
| FOntanella | Widening rural-urban disparities in youth suicides, United States, 1996-2010 | 2015 | JAMA Pediatrics |
| Forst | More Than Training: Community-Based Participatory Research to Reduce Injuries Among Hispanic Construction Workers | 2013 | American Journal of Industrial Medicine |
| Forst et al | Spatial clustering of occupational injuries in communities | 2015 | American Journal of Public Health |
| Fortin | Privately insured medical patients are more likely to have a head CT | 2016 | Emergency Radiology |
| Frank et al | Health care access and health care workforce for immigrant workers in the agriculture, forestry, and fisheries sector in the southeastern US | 2013 | American Journal of Industrial Medicine |
| Freburger | Postacute rehabilitation care for hip fracture: who gets the most care? | 2012 | Journal of the American Geriatrics Society |
| Freeman JJ | A comparison between non-powder gun and powder-gun injuries in a young pediatric population | 2017 | Injury |
| Frencher | A Comparative Analysis of Serious Injury and Illness Among Homeless and Housed Low Income Residents of New York City | 2010 | Journal of Trauma-Injury Infection and Critical Care |
| Friedman | Ethnic disparities in traumatic occupational injury | 2008 | Journal of Occupational and Environmental Medicine |
| Friedman | Analysis of ethnic disparities in workers’ compensation claims using data linkage | 2012 | Journal of Occupational and Environmental Medicine |
| Friedson | Violence and Neighborhood Disadvantage after the Crime Decline | 2015 | Annals of the American Academy of Political and Social Science |
| Fuentes | Functional outcomes during inpatient rehabilitation for American Indian and Alaska Native children with traumatic brain injury | 2016 | Journal of Pediatric Rehabilitation Medicine |
| Fuentes MM | Auditing Access to Outpatient Rehabilitation Services for Children With Traumatic Brain Injury and Public Insurance in Washington State | 2017 | Archives of Physical Medicine and Rehabilitation |
| Fyffe | Racial and ethnic disparities in functioning at discharge and follow-up among patients with motor complete spinal cord injury | 2014 | Archives of Physical Medicine and Rehabilitation |
| Fyffe DC | Vulnerable Groups Living with Spinal Cord Injury | 2011 | Top Spinal Cord Inj Rehabil |
| Garcia AN | Seat belt use among American Indians/Alaska Natives and non-Hispanic whites | 2007 | American Journal of Preventive Medicine |
| García-España | Safety belt laws and disparities in safety belt use among US high-school drivers | 2012 | American Journal of Public Health |
| Gary | Differences in employment outcomes 10 years after traumatic brain injury among racial and ethnic minority groups | 2010 | Journal of Vocational Rehabilitation |
| Gary | Racial differences in employment outcomes after traumatic brain injury at 1, 2, and 5 years post-injury | 2009 | Archives of Physical Medicine and Rehabilitation |
| Geng | Racial-Ethnic Differences in Fall Prevalence among Older Women: A Cross-Sectional Survey Study | 2017 | BMC Geriatrics |
| Gerry | Uninsured status may be more predictive of outcomes among the severely injured than minority race | 2016 | Injury |
| Ghafoori B | Gender Disparities in the Mental Health of Urban Survivors of Trauma | 2013 | Journal of Aggression, Maltreatment, and Trauma |
| Gielen | If you build it, will they come? Using a mobile safety centre to disseminate safety information and products to low-income urban families | 2009 | Injury Prevention |
| Giladi | The Effect of Medicaid Expansion on Delivery of Finger and Thumb Replantation Care to Medicaid Beneficiaries and the Uninsured | 2015 | Plastic and Reconstructive Surgery |
| Gilchrist | Racial and ethnic disparities in fatal unintentional drowning among persons less than 30 years of age United States, 1999-2010 | 2014 | Journal of Safety Research |
| Girasek | An Exploratory Study of the Relationship Between Socioeconomic Status and Motor Vehicle Safety Features | 2010 | Traffic Injury Prevention |
| Glance | Trends in racial disparities for injured patients admitted to trauma centers | 2013 | Health Services Research |
| Godat et al | Do trauma stomas ever get reversed? | 2014 | Journal of the American College of Surgeons |
| Goldstein | Medicaid-based child restraint system disbursement and education and the vaccines for children program: comparative cost-effectiveness | 2008 | Ambulatory Pediatrics |
| Goldstein et al | Explaining regional disparities in traffic mortality by decomposing conditional probabilities | 2011 | Injury Prevention |
| Goldweber | Examining associations between race, urbanicity, and patterns of bullying involvement | 2013 | Journal of Youth and Adolescence |
| Goodenow | Sexual orientation trends and disparities in school bullying and violence-related experiences, 1999–2013 | 2016 | Psychology of Sexual Orientation and Gender Diversity |
| Graham | Race/ethnicity and outcomes following inpatient rehabilitation for hip fracture | 2008 | The Journals of Gerontology Series A |
| Graif | Neighborhood isolation in Chicago: Violent crime effects on structural isolation and homophily in inter-neighborhood commuting networks | 2017 | Social Networks |
| Gray | Suicide in Indian country: The continuing epidemic in rural Native American communities | 2014 | Journal of Rural Mental Health |
| Greene et al | Insurance status is a potent predictor of outcomes in both blunt and penetrating trauma | 2010 | American Journal of Surgery |
| Griffiths | Race, Space, and the Spread of Violence Across the City | 2013 | Social Problems |
| Groah | Spinal cord injury and aging: challenges and recommendations for future research | 2012 | American Journal of Physical Medicine and Rehabilitation |
| Groah | Spinal Cord Injury and Aging Challenges and Recommendations for Future Research | 2012 | American Journal of Physical Medicine & Rehabilitation |
| Groah | Disparities in wheelchair procurement by payer among people with spinal cord injury | 2014 | Physical Medicine and Rehabilitation |
| Grubesic | Comparative modeling approaches for understanding urban violence | 2012 | Social Science Research |
| Grundle | Effect of insurance status on the rate of surgery following a meniscal tear | 2010 | Orthopaedic Journal of Sports Medicine |
| Grzywacz | Work organization and musculoskeletal health: clinical findings from immigrant Latino poultry processing and other manual workers | 2012 | American Journal of Public Health |
| Grzywacz | Using lay health promoters in occupational health: outcome evaluation in a sample of Latino poultry-processing workers | 2009 | New Solutions |
| Gu et al | Racial and health insurance disparities of inpatient spine augmentation of osteoporotic vertebral fractures from 2005 to 2010 | 2014 | American Journal of Neuroradiology |
| Guerrero | Striving for a culturally responsive process in training health professionals on Asian American and Pacific Islander youth violence prevention | 2009 | Aggression and Violent Behavior |
| Gulack | Inequalities in the use of helmets by race and payer status among pediatric cyclists | 2015 | Surgery |
| Gupta | The association between community crime and childhood asthma prevalence in Chicago | 2010 | Annals of Allergy Asthma & Immunology |
| Gurien | An evaluation of trauma outcomes related to insurance status in patients requiring prehospital helicopter transport | 2015 | Prehospital and Disaster Medicine |
| Hadden | An authentic discourse: Recentering race and racism as factors that contribute to police violence against unarmed Black or African American men | 2016 | Journal of Human Behavior in the Social Environment |
| Hagenruch | Disparities in child access to emergency care for acute oral injury | 2011 | Pediatrics |
| Haider | Race and insurance status as risk factors for trauma mortality | 2008 | Archives of Surgery |
| Haider | Females have fewer complications and lower mortality following traumathan similarly injured males: a risk adjusted analysis of adults in the National Trauma Data Bank | 2009 | Surgery |
| Haider | Association between hospitals caring for a disproportionately high percentage of minority trauma patients and increased mortality: a nationwide analysis of 434 hospitals | 2012 | Archives of Surgery |
| Haider | Mortality in adolescent girls vs boys - Following traumatic shock an analysis of the national pediatric trauma registry | 2007 | Archives of Surgery |
| Haider | Unconscious race and class bias: its association with decision making by trauma and acute care surgeons | 2014 | Journal of Trauma and Acute Care Surgery |
| Haider | Unconscious Race and Social Class Bias Among Acute Care Surgical Clinicians and Clinical Treatment Decisions | 2015 | JAMA Surgery |
| Haider AH | Black children experience worse clinical and functional outcomes after traumatic brain injury: an analysis of the National Pediatric Trauma Registry | 2007 | Journal of Trauma |
| Haider et al | Minority trauma patients tend to cluster at trauma enters with worse-than-expected motality: can this phenomenon help explain racial disparities in trauma outcomes? | 2013 | Annals of Surgery |
| Haider et al | Disparities in trauma care and outcomes in the United States: a systematic review and meta-analysis | 2013 | Journal of Trauma and Acute Care Surgery |
| Haines KL | Socioeconomic disparities in the thoracic trauma population | 2017 | Journal of Surgical Research |
| Hakmeh et al | Effect of race and insurance on outcome of pediatric trauma | 2010 | Academic Emergency Medicine |
| Hamilton | The association of insurance status on the probability of transfer for pediatric trauma patients | 2016 | Journal of Pediatric Surgery |
| Hanna | Fatal crash involvement of unlicensed young drivers: County level differences according to material deprivation and urbanicity in the United States | 2012 | Accident Analysis and Prevention |
| Hansen L | Outpatient follow-up after traumatic injury: Challenges and opportunities | 2014 | Journals of Emergencies, Trauma, and Shock |
| Harper | Trends in Socioeconomic Inequalities in Motor Vehicle Accident Deaths in the United States, 1995-2010 | 2015 | American Journal of Epidemiology |
| Harris | Latino immigration and White, Black, and Latino violent crime: A comparison of traditional and non-traditional immigrant destinations | 2013 | Social Science Research |
| Harris | Hispanic Immigration and Black Violence at the Macro-Level: Examining the Conditioning Effect of Victim Race/Ethnicity | 2015 | Sociological Forum |
| Harris | Homicide as a medical outcome: racial disparity in deaths from assault in US Level I and II trauma centers | 2012 | Journal of Trauma and Acute Care Surgery |
| Harrison et al | Work related injury among aging women | 2013 | Policy, Politics, and Nursing Practice |
| Hart | Racial differences in caregiving patterns, caregiver emotional function, and sources of emotional support following traumatic brain injury | 2007 | The Journal of Head Trauma Rehabilitation |
| Haskins et al | Racial disparities in survival among injured drivers | 2013 | American Journal of Epidemiology |
| He | Disparities in Emergency Department Wait Time Among Patients With Traumatic Brain Injury | 2015 | Annals of Emergency Medicine |
| Heerman | Racial and Ethnic Differences in Injury Prevention Behaviors Among Caregivers of Infants | 2016 | American Journal of Preventive Medicine |
| Heerman | Health Literacy and Injury Prevention Behaviors Among Caregivers of Infants | 2014 | American Journal of Preventive Medicine |
| Heffernan | Impact of Socioethnic Factors on Outcomes Following Traumatic Brain Injury | 2011 | Journal of Trauma-Injury Infection and Critical Care |
| Hendrix | Influence of race and neighborhood on the risk for and outcomes of burns in the elderly in North Carolina | 2011 | Burns |
| Herne MA | Homicide Among American Indians/Alaska Natives, 1999-2009: Implications for Public Health Interventions | 2016 | Public Health Reports |
| Hicks et al | Association between race and age in survival after trauma | 2014 | JAMA Surgery |
| Hicks et al | Explaining the paradoxical age-based racial disparities in survival after trauma: the role of the treating facility | 2015 | Annals of Surgery |
| Hicks et al | Racial disparities after vascular trauma are age-dependent | 2016 | Journal of Vascular Surgery |
| Hipp | Intergroup and intragroup violence: Is violent crime an expression of group conflict or social disorganization? | 2009 | Criminology: An Interdisciplinary Journal |
| Hirschfield | Lethal policing: Making sense of American exceptionalism | 2015 | Sociological Forum |
| Hollomotz | Disability, oppression and violence: Towards a sociological explanation | 2013 | Sociology |
| Holst | Undertriage of Trauma-Related Deaths in US Emergency Departments | 2016 | The Western Journal of Emergency Medicine |
| Hoopes | Uncovering a missing demographic in trauma registries: epidemiology of trauma among American Indians and Alaska Natives in Washington State | 2015 | Injury Prevention |
| Hsia et al | Disparities in trauma center access despite increasing utilization: data from California, 1999 to 2006 | 2010 | Journal of Trauma |
| Hu | Reducing black/white disparity: changes in injury mortality in the 15-24 year age group, United States, 1999-2005 | 2008 | Injury Prevention |
| Huang | Association Between Insurance and Transfer of Injured Children From Emergency Departments | 2017 | Pediatrics |
| Huang et al | Insurance disparities in the outcomes of spinal cord stimulation surgery | 2013 | Neuromodulation |
| Hubbard | The decrease in the unintentional injury mortality disparity between American Indians/Alaska Natives and non-American Indians/Alaska Natives in New Mexico, 1980 to 2009 | 2013 | American Journal of Public Health |
| Hughey | The Five I's of Five-O: Racial Ideologies, Institutions, Interests, Identities, and Interactions of Police Violence | 2015 | Critical Sociology |
| Hulland | Socioeconomic Status and Non-Fatal Adult Injuries in Selected Atlanta (Georgia USA) Hospitals | 2017 | Prehospital and Disaster Medicine |
| Humensky | Life is precious: Reducing suicidal behavior in Latinas | 2013 | Ethnicity and Inequalities in Health and Social Care |
| Hwang | Social inequalities in child pedestrian traffic injuries: Differences in neighborhood built environments near schools in Austin, TX, USA | 2017 | Journal of Transport & Health |
| Irizarry CR | Prevalence and ethnic/racial disparities in the distribution of pediatric injuries in South Florida: implications for the development of community prevention programs | 2017 | Injury Epidemiology |
| Irwin | Fighting for Her Honor: Girls' Violence in Distressed Communities | 2012 | Feminist Criminology |
| Ismail | Impact of Video Discharge Instructions for Pediatric Fever and Closed Head Injury from the Emergency Department | 2016 | Journal of Emergency Medicine |
| Isom | An air of injustice? An integrated approach to understanding the link between police injustices and neighborhood rates of violence | 2016 | Journal of Ethnicity in Criminal Justice |
| Jamison | Preliminary investigation of longitudinal sociodemographic, injury and psychosocial characteristics in a group of non-English speaking Latinos with brain injury | 2012 | Brain Injury |
| Janus | Hospital Discharge Destinations for Hispanic and Non-Hispanic White Patients Treated for Traumatic Brain Injury | 2013 | Journal of Trauma Nursing |
| Jarman et al | Rural risk: geographic disparities in trauma mortality | 2016 | Surgery |
| Jerrett | Safe Routes to Play? Pedestrian and Bicyclist Crashes Near Parks in Los Angeles | 2016 | Environmental Research |
| Jesin | Predictors of mortality, hospital utilization, and the role of race in outcomes in head and neck trauma | 2016 | Oral Surgery, Oral Medicine, Oral Pathology, and Oral Radiology |
| Jetelina | The Association Between Familial Homelessness, Aggression, and Victimization Among Children | 2016 | Journal of Adolescent Health |
| Jimenez | Functional independence after inpatient rehabilitation for traumatic brain injury among minority children and adolescents | 2015 | Archives of Physical Medicine and Rehabilitation |
| Jimenez | Utilization of mental health services after mild pediatric traumatic brain injury | 2017 | Pediatrics |
| Jimenez et al | Outpatient Rehabilitation for Medicaid-Insured Children Hospitalized With Traumatic Brain Injury | 2016 | Pediatrics |
| Johnson | Why Us? Toward an Understanding of Bisexual Women's Vulnerability for and Negative Consequences of Sexual Violence | 2017 | Journal of Bisexuality |
| Juárez-Carrillo | Applying learning theory to safety and health training for hispanic immigrant dairy workers | 2017 | Health Promotion Practice |
| Kaba | Traumatic brain injury among newly admitted adolescents in the New York city jail system | 2014 | Journal of Adolescent Health |
| Kalesan | The Hidden Epidemic of Firearm Injury: Increasing Firearm Injury Rates During 2001-2013 | 2017 | American Journal of Epidemiology |
| Kaminski | Assessing the County-Level Structural Covariates of Police Homicides | 2008 | Homicide Studies |
| Kane | Racial/Ethnic and Insurance Status Disparities in Discharge to Posthospitalization Care for Patients With Traumatic Brain Injury | 2014 | Journal of Head Trauma Rehabilitation |
| Karb | County Poverty Concentration and Disparities in Unintentional Injury Deaths: A Fourteen-Year Analysis of 16 Million US Fatalities | 2016 | PLOS One |
| Karriker-Jaffe | Examining how neighborhood disadvantage influences trajectories of adolescent violence: a look at social bonding and psychological distress | 2011 | Journal of School Health |
| Kendrick | Does targeting injury prevention towards families in disadvantaged areas reduce inequalities in safety practices? | 2009 | Health Education Research |
| Kendrick | Preventing childhood falls at home - Meta-analysis and meta-regression | 2008 | American Journal of Preventive Medicine |
| Kent | Killings of police in US cities since 1980: An examination of environmental and political explanations | 2010 | Homicide Studies: An Interdisciplinary & International Journal |
| Kerr | Racial/ethnic disparities in the risk of injury related to the frequency of heavy drinking occasions | 2015 | Alcohol and Alcoholism |
| Kia-Keating | Using community-based participatory research and human-centered design to address violence-related health disparities among Latino/a youth | 2017 | Family & Community Health |
| Kiely DK | Fall Risk is Not Black and White | 2015 | Journal of health disparities, research, and practice |
| Kim | The associations between US state and local social spending, income inequality, and individual all-cause and cause-specific mortality: The National Longitudinal Mortality Study | 2016 | Preventive Medicine |
| Kisser | Lifetime prevalence of traumatic brain injury in a demographically diverse community sample | 2017 | Brain Injury |
| Kodadek et al | Undertriage of older trauma patients: is this a national phenomen? | 2015 | Journal of Surgical Research |
| Kolakowsky | Acceptance rates in state-federal vocational rehabilitation of clients with brain injury: Is racial disparity an issue? | 2010 | Brain Injury |
| Koroukian | Analysis of injury- and violence-related fatalities in the Ohio Medicaid population: Identifying opportunities for prevention | 2007 | Journal of Trauma-Injury Infection and Critical Care |
| Kraemer | Disparities in road crash mortality among pedestrians using wheelchairs in the USA: results of a capture-recapture analysis | 2015 | Bmj Open |
| Kraemer JD | Bicycle helmet laws and persistent racial and ethnic helmet use disparities among urban high school students: a repeated cross-sectional analysis | 2016 | Injury Epidemiology |
| Krause | Race-Ethnicity, Education, and Employment After Spinal Cord Injury | 2010 | Rehabilitation Counseling Bulletin |
| Krause | Emergency room visits and hospitalizations among participants with spinal cord injury | 2015 | NeuroRehabilitation |
| Krause | Disparities in subjective well-being, participation, and health after spinal cord injury: a 6-year longitudinal study | 2009 | NeuroRehabilitation |
| Krivo | Segregation, Racial Structure, and Neighborhood Violent Crime | 2009 | American Journal of Sociology |
| Krivo LJ | Patterns of local segregation: Do they matter for neighborhood crime? | 2015 | Social Science Research |
| Kroshus E | Disparities in Athletic Trainer Staffing in Secondary School Sport: Implications for Concussion Identification | 2017 | Clinical Journal of Sports Medicine |
| Kwon SC | Racial and Ethnic Difference in Falls Among Older Adults: Results from the California Health Interview Survey | 2017 | Journal of Racial and Ethnic Health Disparities |
| Lad et al | Raial disparities in outcomes after spinal cord injury | 2013 | Journal of Neurotrauma |
| Ladha | Factors Affecting the Likelihood of Presentation to the Emergency Department of Trauma Patients After Discharge | 2011 | Annals of Emergency Medicine |
| LaFree | Still separate and unequal? A city-level analysis of the Black-White gap in homicide arrests since 1960 | 2010 | American Sociological Review |
| Lale | Undertriage of Firearm-Related Injuries in a Major Metropolitan Area | 2017 | Jama Surgery |
| Landy | Hispanic ethnicity and unintentional injury mortality in the elderly | 2011 | Journal of Surgical Research |
| Landy | Hispanic ethnicity and fatal fall risk: Do age, gender, and community modify the relationship? | 2012 | Journal of Surgical Research |
| Le | Acculturative dissonance, ethnic identity, and youth violence | 2008 | Cultural Diversity and Ethnic Minority Psychology |
| Le | Risks of non-familial violent physical and emotional victimization in four Asian ethnic groups | 2009 | Journal of Immigrant and Minority Health |
| Lee | Regional variation in the effect of structural factors on homicide in rural areas | 2008 | Social Science Journal |
| Lee et al | Are the racial disparities in the use of retraints and outcomes in children after motor vehicl crashes? | 2012 | Journal of Pediatric Surgery |
| Lehavot | Abuse, mastery, and health among lesbian, bisexual, and two-spirit American Indian and Alaska Native women | 2009 | Cultural Diversity and Ethnic Minority Psychology |
| Lehmann | The impact of advanced age on trauma triage decisions and outcomes: a statewide analysis | 2009 | American Journal of Surgery |
| Lei | Gender equality and violent behavior: How neighborhood gender equality influences the gender gap in violence | 2014 | Violence and Victims |
| Leonhard MJ | Urban/Rural disparities in Oregon pediatric traumatic brain injury | 2015 | Injury Epidemiology |
| Lequerica | Comparison of FIM (TM) communication ratings for English versus non-English speakers in the traumatic brain injury model systems (TBIMS) national database | 2015 | Brain Injury |
| Lesser | Latino adolescent mothers and fathers in south Texas managing violence | 2010 | Issues in Mental Health Nursing |
| Letourneau | Ride Safe: A child passenger safety program for American Indian/Alaska native children | 2008 | Maternal and Child Health Journal |
| Leukhardt | Follow-up disparities after trauma: a real problem for outcomes research | 2010 | American Journal of Surgery |
| Levine | Infant Deaths and Mortality from Gun Violence: Causal or Casual? | 2017 | Journal of the National Medical Association |
| Levine | Sexual Violence Among Middle School Students: The Effects of Gender and Dating Experience | 2017 | Journal of Interpersonal Violence |
| Levy | When love meets hate: The relationship between state policies on gay and lesbian rights and hate crime incidence | 2017 | Social Science Research |
| Liebman | Occupational health policy and immigrant workers in the agriculture, forestry, and fishing sector | 2013 | American Journal of Industrial Medicine |
| Light | Explaining the gaps in white, black, and Hispanic violence since 1990: Accounting for immigration, incarceration, and inequality | 2016 | American Sociological Review |
| Like | Urban inequality and racial differences in risk for violent victimization | 2011 | Crime & Delinquency |
| Like-Haislip | Routine inequality: Violent victimization at the intersection of race and ethnicity among females | 2011 | Violence and Victims |
| Lipsky AM | A comparison of rural versus urban trauma care | 2014 | Journals of Emergencies, Trauma, and Shock |
| Liu LH | Racial and ethnic differences in hip fracture outcomes in men | 2017 | The American Journal of Managed Care |
| Lo | Racial/ethnic differences in hip and diaphyseal femur fractures | 2014 | Osteoporosis International |
| Lo | Racial disparities in age at time of homicide victimization: A test of the multiple disadvantage model | 2015 | Journal of Interpersonal Violence |
| Lo et al | The association of race/ethnicity and risk of atypical femur fracture among older women receiving oral bisphosphonate therapy | 2016 | Bone |
| Lockwood | Mapping crime in Savannah - Social disadvantage, land use, and violent crimes reported to the police | 2007 | Social Science Computer Review |
| Logan | Homicides - United States, 2007 and 2009 | 2013 | MMWR Morbidity and Mortality Weekly Report |
| Losonczy et al | The severity of disparity: increasing injury intensity accentuates disparate outcomes following trauma | 2014 | Journal of Health Care for the Poor and Underserved |
| Lotfipour S | Fatality and injury severity of older adult motor vehicle collisions in orange county, california, 1998-2007 | 2013 | The Western Journal of Emergency Medicine |
| Lovegrove | Examination of the predictors of latent class typologies of bullying involvement among middle school students | 2012 | Journal of School Violence |
| Luthra | Economic fluctuation and crime: a time-series analysis of the effects of oil development in the coastal regions of Louisiana | 2007 | Deviant Behavior |
| Lyons | Defending Turf: Racial Demographics and Hate Crime Against Blacks and Whites | 2008 | Social Forces |
| Lytle | The association of multiple identities with self‐directed violence and depression among transgender individuals | 2016 | Suicide and Life-Threatening Behavior |
| Lytle | The influence of intersecting identities on self‐harm, suicidal behaviors, and depression among lesbian, gay, and bisexual individuals | 2014 | Suicide and Life-Threatening Behavior |
| Ma P | An examination of traffic-related traumatic injuries among children at a Level-1 pediatric trauma center, 2005-2014 | 2017 | Journal of Trauma and Acute Care Surgery |
| MacDonald | Are Immigrant Youth Less Violent? Specifying the Reasons and Mechanisms | 2012 | Annals of the American Academy of Political and Social Science |
| MacDonald | The Privatization of Public Safety in Urban Neighborhoods: Do Business Improvement Districts Reduce Violent Crime Among Adolescents? | 2013 | Law & Society Review |
| Macy | Patient- and Community-Level Sociodemographic Characteristics Associated with Emergency Department Visits for Childhood Injury | 2015 | Journal of Pediatrics |
| Macy | Child passenger safety practices in the US: disparities in light of updated recommendations | 2012 | American Journal of Preventive Medicine |
| Macy | Disparities in Age-Appropriate Child Passenger Restraint Use Among Children Aged 1 to 12 Years | 2014 | Pediatrics |
| Mahmoudi | Emergency Department Wait Time and Treatment of Traumatic Digit Amputation: Do Race and Insurance Matter? | 2017 | Plastic and Reconstructive Surgery |
| Mahmoudi | Longitudinal analysis of hospitalization after spinal cord injury: variation based on race and ethnicity | 2014 | Archives of Physical Medicine and Rehabilitation |
| Males | Teenagers’ high arrest rates: Features of young age or youth poverty? | 2014 | Journal of Adolescent Research |
| Mannix | Neuroimaging for Pediatric Head Trauma: Do Patient and Hospital Characteristics Influence Who Gets Imaged? | 2010 | Academic Emergency Medicine |
| Manzo | A Comparison of Risk Factors Associated with Suicide Ideation/Attempts in American Indian and White Youth in Montana | 2015 | Archives of Suicide Research |
| Marin LS | Results of a community-based survey of construction safety climate for Hispanic workers | 2015 | International Journal of Occupational and Environmental Health |
| Marshall GN | Ethnic differences in posttraumatic distress: Hispanics' symptoms differ in kind and degree | 2009 | Journal of Consulting and Clinical Psychology |
| Martin et al | Race disparities in firearm injuries and outcomes among Tennessee children | 2012 | Journal of Pediatric Surgery |
| Martinez | Social disorganization, drug market activity, and neighborhood violent crime | 2008 | Urban Affairs Review |
| Martinez | The Role of Immigration: Race/Ethnicity and San Diego Homicides Since 1970 | 2016 | Journal of Quantitative Criminology |
| Martinez | Language discordance and patient-centered care in occupational therapy: A case study | 2015 | OTJR: Occupation, Participation and Health |
| Mascialino | Objective and subjective assessment of long-term community integration in minority groups following traumatic brain injury | 2009 | Neurorehabilitation |
| Massetti | Preventing Violence Among High-Risk Youth and Communities with Economic, Policy, and Structural Strategies | 2016 | MMWR Morbidity and Mortality Weekly Report |
| Mattocks | Sexual victimization, health status, and VA healthcare utilization among lesbian and bisexual OEF/OIF veterans | 2013 | Journal of General Internal Medicine |
| Maurer Jones | Musculoskeletal healthcare disparities: influence of patient sex, race, equity on utilization of total join arthoplasty | 2014 | Journal of Long-Term Effects of Medical Implants |
| Maybury et al | Pedestrians struck by motor transportationals further worsen race- and insurance-based disparities in trauma outcomes: the case for inner-city pedestrian injury prevention programs | 2010 | Surgery |
| McAndrews | Linking transportation and population health to reduce racial and ethnic disparities in transportation injury: Implications for practice and policy | 2017 | International Journal of Sustainable Transportation |
| McCabe | Community gardens to fight urban youth crime and stabilize neighborhoods | 2014 | International Journal of Child Health and Human Development |
| McCoy | Race-related Healthcare Disparities Among California Workers: Public Health Considerations for Immigration Reform | 2016 | Journal of Emergency Medicine |
| McCutcheon | Treatment biases in traumatic neurosurgical care: a retrospective study of the Nationwide Inpatient Sample from 1998 to 2009 | 2015 | Journal of Neurosurgery |
| McDonald | Community violence exposure and positive youth development in urban youth | 2011 | Journal of Community Health |
| McNulty | Neighborhood disadvantage and verbal ability as explanations of the black–white difference in adolescent violence: Toward an integrated model | 2013 | Crime & Delinquency |
| McQuiestion | Insurance status and race affect treatment and outcome of traumatic brain injury | 2016 | Journal of Surgical Research |
| Meade | Perceptions of Provider Education and Attitude by Individuals with Spinal Cord Injury: Implications for Health Care Disparities | 2011 | Spinal Cord Injury Rehabilitation |
| Meagher | Racial and ethnic disparities in discharge to rehabilitation following traumatic brain injury | 2015 | Journal of Neurosurgery |
| Mello | Injuries in youth football: national emergency department visits during 2001-2005 for young and adolescent players | 2009 | Academic Emergency Medicine |
| Menendez | Work-Related Violent Deaths in the US Taxi and Limousine Industry 2003 to 2013 Disparities Within a High-Risk Working Population | 2017 | Journal of Occupational and Environmental Medicine |
| Menéndez | Disparities in work-related homicide rates in selected retail industries in the United States, 2003–2008 | 2013 | Journal of Safety Research |
| Menzel NN | Social marketing to plan a fall prevention program for Latino construction workers | 2012 | American Journal of Industrial Medicine |
| Messner | Revisiting the Quality and Use of Race-Specific Homicide Data: Exploring Substantive Implications | 2014 | Homicide Studies |
| Metcalfe et al | Access to post-discharge inpatient care after low limb trauma | 2016 | Journal of Surgical Research |
| Michael | Women are less likely than men to receive prehospital analgesia for isolated extremity injuries | 2007 | American Journal of Emergency Medicine |
| Mikhail | The Association of Race, Socioeconomic Status, and Insurance on Trauma Mortality | 2016 | Journal of Trauma Nursing |
| Miller | Violence against urban African American girls challenges for feminist advocacy | 2008 | Journal of Contemporary Criminal Justice |
| Miller | Suicide mortality in the United States: The importance of attending to method in understanding population-level disparities in the burden of suicide | 2012 | Annual Review of Public Health |
| Miller | Exposure to partner, family, and community violence: Gang-affiliated Latina women and risk of unintended pregnancy | 2012 | Journal of Urban Health-Bulletin of the New York Academy of Medicine |
| Millham | Are there racial disparities in trauma care? | 2009 | World Journal of Surgery |
| Missios et al | The association of insurance status and race with the procedural volume of traumatic brain injury patients | 2016 | Injury |
| Mohr NM | Telemedicine Use Decreases Rural Emergency Department Length of Stay for Transferred North Dakota Trauma Patients | 2017 | Telemedicine and e-Health |
| Moore | Availability of Outpatient Rehabilitation Services for Children After Traumatic Brain Injury: Differences by Language and Insurance Status | 2016 | American Journal of Physical Medicine & Rehabilitation |
| Moreira et al | Racial differences in treatment approaches and mortality following arterial trauma | 2015 | Vascular and Endovascular Surgery |
| Murphy | Unintentional injury mortality among American Indians and Alaska Natives in the United States, 1990-2009 | 2014 | American Journal of Public Health |
| Mustanski | A syndemic of psychosocial health disparities and associations with risk for attempting suicide among young sexual minority men | 2014 | American Journal of Public Health |
| Myaskovsky L | How Are Race, Cultural, and Psychosocial Factors Associated With Outcomes in Veterans With Spinal Cord Injury? | 2017 | Archives of Physical Medicine and Rehabilitation |
| Natale | Relationship of Physician-identified Patient Race and Ethnicity to Use of Computed Tomography in Pediatric Blunt Torso Trauma | 2016 | Academic Emergency Medicine |
| Natale | Cranial Computed Tomography Use Among Children With Minor Blunt Head Trauma | 2012 | Archives of Pediatrics & Adolescent Medicine |
| Nery-Hurwit | Stakeholder evaluation of an online program to promote physical activity and workplace safety for individuals with disability | 2017 | Evaluation and Program Planning |
| Neuman | Race and patient preferences for hip fracture care | 2013 | Journal of the American Geriatrics Society |
| Neuman et al | Breakout session: Sex/Gender and racial/ethnic disparities in the care of osteoporosis and fragility fractures | 2011 | Clinical Orthopedics and Related Research |
| Neuman et al | nonoperative care for hip fractures in the elderly: the inflluence of race, income, and comorbidities | 2010 | Medical Care |
| Neuner JM | Racial and socioeconomic disparities in bone density testing before and after hip fracture | 2007 | Journal of General Internal Medicine |
| Ngo | Stressful life events, culture, and violence | 2007 | Journal of Immigrant and Minority Health |
| Nguyen | Variations in Utilization of Inpatient Rehabilitation Services among Pediatric Trauma Patients | 2017 | Journal of Pediatrics |
| Nguyen-Oghalai | Disparities in utilization of outpatient rehabilitative care following hip fracture hospitalization with respect to race and ethnicity | 2009 | Archives of Physical Medicine and Rehabilitation |
| Nicholson | Disparities in work-related injuries associated with worker compensation coverage status | 2008 | American Journal of Industrial Medicine |
| Nicholson-Crotty | Will More Black Cops Matter? Officer Race and Police-Involved Homicides of Black Citizens | 2017 | Public Administration Review |
| Nicolaidis | "You don't go tell White people nothing": African American women's perspectives on the influence of violence and race on depression and depression care | 2010 | American Journal of Public Health |
| Niemeier | Toward improved rehabilitation services for ethnically diverse survivors of traumatic brain injury | 2007 | Journal of Head Trauma Rehabilitation |
| Nirula | Inequity of rehabilitation services after traumatic injury | 2009 | Journal of Trauma and Acute Care Surgery |
| Nweze | Demographic and socioeconomic factors influencing disparitiesin prevalence of alcohol-related injury among underserved trauma patients in a safety-net hospital | 2016 | Injury |
| O'Flaherty | Homicide in black and white | 2010 | Journal of Urban Economics |
| Oh | Fatal Pediatric Motor Vehicle Crashes on US Native American Indian Lands Compared to Adjacent Non-Indian Lands: Restraint Use and Injury by Driver, Vehicle, Roadway and Crash Characteristics | 2017 | International Journal of Environmental Research and Public Health |
| Oshri | Developmental Trajectories of Substance Use Among Sexual Minority Girls: Associations With Sexual Victimization and Sexual Health Risk | 2014 | Journal of Adolescent Health |
| Osler | Survival Rates in Trauma Patients Following Health Care Reform in Massachusetts | 2015 | JAMA Surgery |
| Osler | Trauma care does not discriminate: The association of race and health insurance with mortality following traumatic injury | 2015 | Journal of Trauma and Acute Care Surgery |
| Overton | The Hispanic paradox: does it exist in the injured? | 2015 | American Journal of Surgery |
| Oyetunji et al | Profiling the ethnic characteristics of domestic injuries in children younger than age 5 years | 2012 | American Journal of Surgery |
| Pabayo | The role of neighborhood income inequality in adolescent aggression and violence | 2014 | Journal of Adolescent Health |
| Panikkar | Occupational health and safety experiences among self-identified immigrant workers living or working in Somerville, MA by ethnicity, years in the US, and English proficiency | 2012 | International Journal of Environmental Research and Public Health |
| Panikkar | Occupational health outcomes among self-identified immigrant workers living and working in Somerville, Massachusetts | 2013 | Journal of Immigrant and Minority Health |
| Papachristos | The Corner and the Crew: The Influence of Geography and Social Networks on Gang Violence | 2013 | American Sociological Review |
| Papachristos | Tragic, but not random: the social contagion of nonfatal gunshot injuries | 2015 | Social Science & Medicine |
| Papachristos | More Coffee, Less Crime? The Relationship between Gentrification and Neighborhood Crime Rates in Chicago, 1991 to 2005 | 2011 | City & Community |
| Pappadis | Effectiveness of an Educational Intervention on Reducing Misconceptions Among Ethnic Minorities With Complicated Mild to Severe Traumatic Brain Injury | 2017 | Archives of Physical Medicine and Rehabilitation |
| Parham-Payne | The Role of the Media in the Disparate Response to Gun Violence in America | 2014 | Journal of Black Studies |
| Parker | Intersections of Race, Gender, Disadvantage, and Violence: Applying Intersectionality to the Macro-Level Study of Female Homicide | 2015 | Justice Quarterly |
| Parker | The Changing Urban Landscape: Interconnections Between Racial/Ethnic Segregation and Exposure in the Study of Race-Specific Violence Over Time | 2015 | American Journal of Public Health |
| Parrish | Understanding our patient's disparities and needs: a brief report in the use of a Spanish lesson to increase the comfort level during trauma situations | 2014 | American Surgeon |
| Pavkov et al | Tribal youth victimization and delinquency: analysis of Youth Risk Behavior Surveillance Survey data | 2010 | Cultural diversity and ethnic minority psychology |
| Peguero | Backlash for breaking racial and ethnic breaking stereotypes: Adolescent school victimization across contexts | 2016 | Journal of Interpersonal Violence |
| Peguero AA. | Violence, schools, and dropping out: racial and ethnic disparities in the educational consequence of student victimization | 2011 | Journal of Interpersonal Violence |
| Pelletier | Learning in harm’s way: Neighborhood violence, inequality, and American schools | 2017 | Annals of the American Academy of Political and Social Science |
| Pena | Co-occurring risk behaviors among White, Black, and Hispanic US high school adolescents with suicide attempts requiring medical attention, 1999-2007: Implications for future prevention initiatives | 2012 | Social Psychiatry and Psychiatric Epidemiology |
| Penrod | The association of race, gender, and comorbidity with mortality and function after hip fracture | 2008 | The Journals of Gerontology Series A |
| Periyanayagam U | Predictors of assault among urban female trauma patients | 2012 | Journal of Emergiencies, Trauma, and Shock |
| Perron | Prevalence and correlates of traumatic brain injury among delinquent youths | 2008 | Criminal Behaviour and Mental Health |
| Peskin | Bullying and victimization and internalizing symptoms among low‐income Black and Hispanic students | 2007 | Journal of Adolescent Health |
| Peterson | Segregated spatial locations, race-ethnic composition, and neighborhood violent crime | 2009 | Annals of the American Academy of Political and Social Science |
| Peterson TH | Partnering with youth organizers to prevent violence: an analysis of relationships, power, and change | 2010 | Progress in Community Health Partnerships: Research, Education, and Action |
| Peura C | Evaluating adverse rural crash outcomes using the NHTSA State Data System | 2015 | Accident Analysis and Prevention |
| Piatt | Pediatric spinal injury in the US: epidemiology and disparities | 2015 | Journal of Neurosurgery: Pediatrics |
| Piatt | Hospital care of childhood traumatic brain injury in the United States, 1997-2009: a neurosurgical perspective Clinical article | 2012 | Journal of Neurosurgery-Pediatrics |
| Pickham | Payer status is associated with the use of prophylactic inferior vena cava filter in high-risk trauma patients | 2012 | Surgery |
| Piontkowski | Reducing Motor vehicle-Related Injuries at an Arizona Indian Reservation: Ten Years of Application of Evidence-Based Strategies | 2015 | Global Health Science Practice |
| Pizarro | An Examination of the Covariates of Female Homicide Victimization and Offending | 2010 | Feminist Criminology |
| Plurad | The association of race and survival from sepsis after injury | 2010 | The American Journal of Surgery |
| Pompeii | Surveillance of musculoskeletal injuries and disorders in a diverse cohort of workers at a tertiary care medical center | 2008 | American Journal of Industrial Medicine |
| Pransky | Geographic Variation in Early MRI for Acute Work-Related Low Back Pain and Associated Factors | 2015 | Spine |
| Pressley | Epidemiology of bone fracture across the age span in blacks and whites | 2011 | Journal of Trauma |
| Pressley | National injury-related hospitalizations in children: Public versus private expenditures across preventable injury mechanisms | 2007 | Journal of Trauma-Injury Infection and Critical Care |
| Pressley | Motor vehicle occupant injury and related hospital expenditures in children aged 3 years to 8 years covered versus uncovered by booster seat legislation | 2009 | Journal of Trauma |
| Pressley | Race and ethnic differences in a multicenter study of home safety with vouchers redeemable for free safety devices | 2009 | Journal of Trauma |
| Pressley JC | Twenty-year trends in fatal injuries to very young children: the persistence of racial disparities | 2007 | Pediatrics |
| Proctor | Performance of three racial/ethnic groups on two tests of executive function: Clinical implications for traumatic brain injury (TBI) | 2008 | NeuroRehabilitation |
| Putnam-Hornstein | Preventable injury deaths: a population-based proxy of child maltreatment risk inCalifornia | 2012 | Public Health Reports |
| Pyrooz | Structural Covariates of Gang Homicide in Large US Cities | 2012 | Journal of Research in Crime and Delinquency |
| Quandt | Occupational Eye Injuries Experienced by Migrant Farmworkers | 2012 | Journal of Agromedicine |
| Quandt et al | 3D and health disparities: The helth implications of Latino chicken catchers' working conditions | 2013 | American Journal of Industrial Medicine |
| Quillian | Estimating Risk: Stereotype Amplification and the Perceived Risk of Criminal Victimization | 2010 | Social Psychology Quarterly |
| Ramirez | Pediatric Injury Outcomes in Racial/Ethnic Minorities in California Diversity May Reduce Disparity | 2013 | JAMA Surgery |
| Ramirez et al | Can universal coverage eliminate health disparities? Reversal of disparate injury outcomes in elderly insured minorities | 2013 | Journal of Surgical Research |
| Rangel | Alarming trends in the improper use of motor transportational restraints in children: implications for public policy and the development of race-based strategies for improving compliance | 2008 | Journal of Pediatric Surgery |
| Rauscher | Socioeconomic disparities in the prevalence of work-related injuries among adolescents in the United States | 2008 | Journal of Adolescent Health |
| Redelings | Years off Your Life? The Effects of Homicide on Life Expectancy by Neighborhood and Race/Ethnicity in Los Angeles County | 2010 | Journal of Urban Health |
| Redmond | Association of Recent Incarceration With Traumatic Injury, Substance Use-Related Health Consequences, and Health Care Utilization | 2014 | Journal of Addiction Medicine |
| Reed | Experiences of racial discrimination & relation to violence perpetration and gang involvement among a sample of urban African American men | 2010 | Journal of Immigrant and Minority Health |
| Reed CR | Insurance status, mortality, and hospital use among pediatric trauma patients over three decades | 2017 | Journal of Pediatric Surgery |
| Reisner | A compensatory model of risk and resilience applied to adolescent sexual orientation disparities in nonsuicidal self-injury and suicide attempts | 2014 | American Journal of Orthopsychiatry |
| Rhew | Effects of sexual assault on alcohol use and consequences among young adult sexual minority women | 2017 | Journal of Consulting and Clinical Psychology |
| Rivera | Disability After Deployment Injury: Are Women and Men Service Members Different? | 2015 | Clinical Orthopedic and Related Research |
| Roberts | US spousal homicide rates by racial composition of marriage | 2015 | Annals of Epidemiology |
| Roberts et al | Pervasive trauma exposure among US sexual orientation minority adults and risk of posttraumatic stress disorder | 2010 | American Journal of Public Health |
| Robinson | The Effect of Urban Street Gang Densities on Small Area Homicide Incidence in a Large Metropolitan County, 1994-2002 | 2009 | Journal of Urban Health-Bulletin of the New York Academy of Medicine |
| Rockett | Race/ethnicity and potential suicide misclassification: window on a minority suicide paradox? | 2010 | BMC Psychiatry |
| Rogers | Description of the moderate brain injured patient and predictors of discharge to rehabilitation | 2015 | Archives of Physical Medicine and Rehabilitation |
| Roman | Rehabilitation Counselor Competencies When Working With Hispanic/Latino Immigrant Injured Workers: A Delphi Study | 2017 | Rehabilitation Research, Policy, and Education |
| Rood | Predictors of suicidal ideation in a statewide sample of transgender individuals | 2015 | LGBT Health |
| Rosen | Downwardly mobile: the accidental cost of being uninsured | 2009 | Archives of Surgery |
| Rosen | Lack of insurance negatively affects trauma mortality in US children | 2009 | Journal of Pediatric Surgery |
| Rosenbaum | Prevalence of epicondylitis, rotator cuff syndrome, and low back pain in Latino poultry workers and manual laborers | 2013 | American Journal of Industrial Medicine |
| Ross | A Multi-Level Bayesian Analysis of Racial Bias in Police Shootings at the County-Level in the United States, 2011-2014 | 2015 | PLoS One |
| Rubin | Racial differences in withdrawal of mechanical ventilation do not alter mortality in neurologically injured patients | 2014 | Journal of Critical Care |
| Russell | Indicators of victimization and sexual orientation among adolescents: Analyses from Youth Risk Behavior Surveys | 2014 | American Journal of Public Health |
| Ryan et al | Delay in hip fracture surgery: an analysis of patient-specific and hospital-specific risk factors | 2015 | Journal of Orthopedic Trauma |
| Ryb Dischinger | Disparities in trauma center access of older injured motor vehical crash occupants | 2011 | The Journal of Trauma: Injury, Infection, and Critical Care |
| Ryu | Individual housing-based socioeconomic status predicts risk of accidental falls among adults | 2017 | Annals of Epidemiology |
| Sabbath | Obscured by administrative data? Racial disparities in occupational injury | 2017 | Scandinavian Journal of Work Environment & Health |
| Sabharwal | Pediatric orthopaedic patients presenting to a university emergency department after visiting another emergency department - Demographics and health insurance status | 2007 | Journal of Pediatric Orthopaedics |
| Sacks et al | Insurance status and hospital discharge disposition after trauma: inequities in access to postacute care | 2011 | Journal of Trauma |
| Saladin | Pressure ulcer prevalence and barriers to treatment after spinal cord injury: comparisons of four groups based on race-ethnicity | 2009 | NeuroRehabilitation |
| Salas-Wright | Trends in Fighting and Violence Among Adolescents in the United States, 2002-2014 | 2017 | American Journal of Public Health |
| Salim | Does insurance status matter at a public, level I trauma center? | 2010 | Journal of Trauma |
| Sander | Relationship of race/ethnicity to caregivers’ coping, appraisals, and distress after traumatic brain injury | 2007 | Neurorehabilitation |
| Sander | Relationship of race/ethnicity and income to community integration following traumatic brain injury: Investigation in a non-rehabilitation trauma sample | 2009 | Neurorehabilitation |
| Saunders | The Relationship of Pressure Ulcers, Race, and Socioeconomic Conditions After Spinal Cord Injury | 2010 | Journal of Spinal Cord Medicine |
| Saunders et al | Gender, race, pain, and subjective well-being of adults with spinal cord injury | 2013 | Journal of Health Care for the Poor and Underserved |
| Sawhney | Impact of Type of Health Insurance on Infection Rates among Young Trauma Patients | 2016 | Surgical Infections |
| Schiraldi et al | Effect of insurance and racial disparities on outcomes in traumatic brain injury | 2015 | Journal of Neurological Surgery |
| Schneider | Beating the weekend trend: increased mortality in older adult traumatic brain injury (TBI) patients admitted on weekends | 2012 | Journal of Surgical Research |
| Schoenfeld | The influence of race and hospital environment on the care of patients with cervical spine fractures | 2016 | Spine Journal |
| Schoenfeld | Patient demographics, insurance status, race, and ethnicity as predictors of morbidity and mortality after spine trauma: a study using the National Trauma Data Bank | 2013 | Spine Journal |
| Scholtes B | Ethical considerations for the design and implementation of child injury prevention interventions: the example of delivering and installing safety equipment into the home | 2017 | Injury Prevention |
| Schwartz | Stability and Change in Female and Male Violence across Rural and Urban Counties, 1981-2006 | 2010 | Rural Sociology |
| Schwebel | Prevalence and Correlates of Firearm Ownership in the Homes of Fifth Graders: Birmingham, AL, Houston, TX, and Los Angeles, CA | 2014 | Health Education & Behavior |
| Scott | Potential impact of Affordable Care Act-related insurance expansion on trauma care reimbursement | 2017 | Journal of Trauma and Acute Care Surgery |
| Scott et al | Counting the lives lost: how many black trauma deaths are attributable to disparities? | 2013 | Journal of Surgical Research |
| Scott et al | Dependent coverage provision led to uneven insurance gains and unchanged mortality rates in young adult trauma patients | 2015 | Health Affairs |
| Scott et al | Racial and Regional disparities in the effect of the affordable care act's dependent coverage provision on young adult trauma patients | 2015 | Journal of the American College of Surgeons |
| Seabury | Racial And Ethnic Differences In The Frequency Of Workplace Injuries And Prevalence Of Work-Related Disability | 2017 | Health Affairs |
| Seabury | Racial Disparities in the Frequency of Workplace Injuries | 2015 | Annals of Emergency Medicine |
| Sears | Trends in the disproportionate burden of work-related traumatic injuries sustained by Latinos | 2012 | Journal of Occupational and Environmental Medicine |
| Sears | Who Pays for Work-Related Traumatic Injuries? Payer Distribution in Washington State by Ethnicity, Injury Severity, and Year (1998-2008) | 2013 | American Journal of Industrial Medicine |
| Sears et al | Disparities in occupational injury hospitalization rates in five states (2003-2009) | 2015 | American Journal of Industrial Medicine |
| Setien | Does Injury Prevention Education Initiate Household Changes in a Spanish-Speaking Minority Population? | 2014 | Journal of Community Health |
| Sewell | The illness associations of police violence: Differential relationships by ethnoracial composition | 2017 | Sociological Forum |
| Shafi | Ethnic disparities in initial management of trauma patients in a nationwide sample of emergency department visits | 2008 | Archives of Surgery |
| Shafi | Ethnic disparities exist in trauma care | 2007 | Journal of Trauma |
| Shafi | Racial disparities in long-term functional outcome after traumatic brain injury | 2007 | Journal of Trauma |
| Shannon | Race, Racial Discrimination, and the Risk of Work-Related Illness, Injury, or Assault: Findings From a National Study | 2009 | Journal of Occupational and Environmental Medicine |
| Sharma M | Universal Health Insurance and its association with long term outcomes in Pediatric Trauma Patients | 2017 | Injury |
| Shetgiri | Parental characteristics associated with bullying perpetration in US children aged 10 to 17 years | 2012 | American Journal of Public Health |
| Shetgiri R | Adolescent fighting: racial/ethnic disparities and the importance of families and schools | 2010 | Academic Pediatrics |
| Shi | Medical expenditures associated with nonfatal occupational injuries among US workers reporting persistent disabilities | 2015 | Disability and Health Journal |
| Shihadeh | Latino Employment and Non-Latino Homicide in Rural Areas: The Implications of US Immigration Policy | 2010 | Deviant Behavior |
| Shihadeh | Latino Immigration, Economic Deprivation, and Violence: Regional Differences in the Effect of Linguistic Isolation | 2010 | Homicide Studies |
| Shihadeh | Latino Employment and Black Violence: The Unintended Consequence of US Immigration Policy | 2010 | Social Forces |
| Shrestha PP | Hispanic construction workers and assertiveness training | 2014 | Work |
| Shuval K | "I live by shooting hill"-a qualitative exploration of conflict and violence among urban youth in New Haven, Connecticut | 2012 | Journal of Health Care for the Poor and Underserved |
| Singer | Insurance- and race-related disparities decrease in elderly trauma patients | 2013 | Journal of Trauma and Acute Care Surgery |
| Singer et al | Insurance-and-race related disparities decrease in elderly trauma patients | 2013 | Journal of Trauma and Acute Care Surgery |
| Smith | Becoming who we are: A theoretical explanation of gendered social structures and social networks that shape adolescent interpersonal aggression | 2009 | Psychology of Women Quarterly |
| Smith | Work-related injuries among commercial janitors in Washington state, comparisons by gender | 2017 | Journal of Safety Research |
| Smith | State-level social capital and suicide mortality in the 50 US states | 2014 | Social Science & Medicine |
| Smith JR | Posttraumatic stress symptoms in context: Examining trauma responses to violent exposures and homicide death among Black males in urban neighborhoods | 2016 | American Journal of Orthopsychiatry |
| Smith JR. | Unequal burdens of loss: examining the frequency and timing of homicide deaths experienced by young Black men across the life course | 2015 | American Journal of Public Health |
| Snyder | Helmet use among Alaskan children involved in off-road motorized transportational crashes | 2014 | International Journal of Circumpolar Health |
| Sonderman | Suicides, Homicides, Accidents, and Other External Causes of Death among Blacks and Whites in the Southern Community Cohort Study | 2014 | PLOS One |
| Sorani | Race\Ethnicity and Outcome After Traumatic Brain Injury at a Single, Diverse Center | 2009 | Journal of Trauma-Injury Infection and Critical Care |
| Sorenson | Gender disparities in injury mortality: consistent, persistent, and larger than you'd think | 2011 | American Journal of Public Health |
| Souza et al | Surveillance of occupational health disparities, challenges and opportunities | 2010 | American Journal of Industrial Medicine |
| Spencer | Exploring the hypothesis of ethnic practice as social capital: Violence among Asian/Pacific Islander youth in Hawaii | 2009 | International Journal of Social Psychiatry |
| Sperry et al | racial disparities and sex-based outcomes differences after severe injury | 2012 | Journal of the American College of Surgeons |
| Spriggs | Adolescent bullying involvement and perceived family, peer and school relations: Commonalities and differences across race/ethnicity | 2007 | Journal of Adolescent Health |
| Squitieri | Patterns of surgical care and health disparities of treating pediatric finger amputation injuries in the United States | 2011 | Journal of the American College of Surgeons |
| Sridharan | Injury and Health Among Children in Vulnerable Families | 2011 | Journal of Trauma-Injury Infection and Critical Care |
| Stanbury Rosenman | Occupational health disparities: a state public health-based approach | 2014 | American Journal of Industrial Medicine |
| Statter et al | Targeting pediatric pedestrian injury prevention efforts: teasing the information through spatial analysis | 2011 | Journal of Trauma |
| Staudenmayer | Ethnic disparities in long-term functional outcomes after traumatic brain injury | 2007 | Journal of Trauma |
| Steege et al | Examining occupational health and safety disparities using national data: a cause for continuing concern | 2014 | American Journal of Industrial Medicine |
| Steffensmeier | Reassessing trends in Black violent crime, 1980–2008: Sorting out the 'Hispanic effect' in uniform crime reports arrests, national crime victimization survey offender estimates, and US prisoner counts | 2011 | Criminology: An Interdisciplinary Journal |
| Steffensmeier | SCOPE AND CONCEPTUAL ISSUES IN TESTING THE RACE-CRIME INVARIANCE THESIS: BLACK, WHITE, AND HISPANIC COMPARISONS | 2010 | Criminology |
| Stempski | Everyone Swims: a community partnership and policy approach to address health disparities in drowning and obesity | 2015 | Health Education & Behavior |
| Stephens et al | Ethnoracial variations in acute PTSD symptoms among hospitalized survivors of traumatic injury | 2010 | Journal of Traumatic Stress |
| Stone | Factors associated with trauma clinic follow-up compliance after discharge: experience at an urban Level I trauma center | 2014 | Journal of Trauma and Acute Care Surgery |
| Stone | Men Who Kill Policemen | 2015 | Violence and Gender |
| Stowell | IMMIGRATION AND THE RECENT VIOLENT CRIME DROP IN THE UNITED STATES: A POOLED, CROSS-SECTIONAL TIME-SERIES ANALYSIS OF METROPOLITAN AREAS | 2009 | Criminology |
| Stowell | Displaced, dispossessed, or lawless? Examining the link between ethnicity, immigration, and violence | 2007 | Aggression and Violent Behavior |
| Strom | The influence of social and economic disadvantage on racial patterns in youth homicide over time | 2007 | Homicide Studies |
| Strong BL | Outcomes of trauma admission for falls: influence of race and age on inhospital and post-discharge mortality | 2016 | American Journal of Surgery |
| Sugarman | Is the enrollment of racial and ethnic minorities in research in the emergency setting equitable? | 2009 | Resuscitation |
| Sullins | Racial/ethnic and socioeconomic disparities in the use of helmets in children involved in bicycle accidents | 2014 | Journal of Pediatric Surgery |
| Sullivan et al | Demographic factors in hip fracture incidence and mortality rates in California, 2000-2011 | 2016 | Journal of Orthopaedic Sugery and Research |
| Sumner | Violence in the United States: Status, challenges, and opportunities | 2015 | JAMA |
| Swisher | If they grow up: Exploring the neighborhood context of adolescent and young adult survival expectations | 2013 | Journal of Research on Adolescence |
| Sykes | Adolescent racial discrimination and parental perceptions of safety in American neighborhoods and schools | 2017 | Sociological Forum |
| Szlachcic Y | Cardiometabolic changes and disparities among persons with spinal cord injury: a 17-year cohort study | 2014 | Top Spinal Cord Inj Rehabil |
| Taghavi | Does payer status matter in predicting penetrating trauma outcomes? | 2012 | Surgery |
| Taiwo | Sex differences in injury patterns among workers in heavy manufacturing | 2009 | American Journal of Epidemiology |
| Tepas | Insurance status, not race, is a determinant of outcomes from vehicular injury | 2011 | Journal of the American College of Surgeons |
| Thomas | Walking away hurt, walking around scared: A cluster analysis of violence exposure among young Black males | 2016 | Journal of Black Psychology |
| Threadcraft | Black women, victimization, and the limitations of the liberal state | 2017 | Theoretical Criminology |
| Tiesman | Occupational and non-occupational injuries in the United States Army: Focus on gender | 2007 | American Journal of Preventive Medicine |
| Tiesman | Suicide in US Workplaces, 2003-2010 A Comparison With Non-Workplace Suicides | 2015 | American Journal of Preventive Medicine |
| Tingey | ENTREPRENEURSHIP EDUCATION: A STRENGTH-BASED APPROACH TO SUBSTANCE USE AND SUICIDE PREVENTION FOR AMERICAN INDIAN ADOLESCENTS | 2016 | American Indian and Alaska Native Mental Health Research |
| Tita | Spatializing the Social Networks of Gangs to Explore Patterns of Violence | 2011 | Journal of Quantitative Criminology |
| Tonozzi | Reported work-related injuries and illnesses among Hispanic workers: Results from an emergency department surveillance system follow-back survey | 2016 | American Journal of Industrial Medicine |
| Tribble | Musculoskeletal pain, depression, and stress among Latino manual laborers in North Carolina | 2016 | Archives of Environmental & Occupational Health |
| Triplett | Zero tolerance, school shootings, and the post-Brown quest for equity in discipline policy: An examination of how urban minorities are punished for White suburban violence | 2014 | Journal of Negro Education |
| Tsai | Racial/ethnic differences in emergency care for joint dislocation in 53 US EDs | 2012 | The American Journal of Emergency Medicine |
| Tsai | Occupational hazards and risks faced by Chinese immigrant restaurant workers | 2007 | Family & Community Health |
| Tsai | Chinese immigrant restaurant workers' injury and illness experiences | 2009 | Archives of Environmental & Occupational Health |
| Tsai JH | Effects of Social Determinants on Chinese Immigrant Food Service Workers' Work Performance and Injuries: Mental Health as a Mediator | 2015 | Journal of Occupational and Environmental Medicine |
| Turanovic | Consequences of Violent Victimization for Native American Youth in Early Adulthood | 2017 | Journal of Youth and Adolescence |
| Ulirsch | No man is an island: Living in a disadvantaged neighborhood influences chronic pain development after motor vehicle collision | 2014 | Pain |
| Ulmer JT | Racial and Ethnic Disparities in Structural Disadvantage and Crime: White, Black, and Hispanic Comparisons | 2012 | Social Science Quarterly |
| Vaca | The Latino adolescent male mortality peak revisited: Attribution of homicide and motor vehicle crash death | 2011 | Injury Prevention |
| Vaca F | US motor vehicle fatality trends in young Latino males | 2009 | Association for the Advancement of Automotive Medicine |
| Velez | NEIGHBORHOOD HOUSING INVESTMENTS AND VIOLENT CRIME IN SEATTLE, 1981-2007 | 2012 | Criminology |
| Vettukattil | Do trauma safety-net hospitals deliver truly safe trauma care? A multilevel analysis of the national trauma data bank | 2011 | Journal of Trauma |
| Vieraitis | Assessing the Impact of Changes in Gender Equality on Female Homicide Victimization: 1980-2000 | 2015 | Crime & Delinquency |
| Vieraitis | Women's status and risk of homicide victimization: An analysis with data disaggregated by victim-offender relationship | 2008 | Homicide Studies: An Interdisciplinary & International Journal |
| Vyas et al | Pediatric facial fractures: current national incidence, distribution, and health care resource use | 2008 | Journal of Craniofacial Surgery |
| Walker | Rates of firearm homicide by Chicago region, age, sex, and race/ethnicity, 2005-2010 | 2016 | Journal of Trauma and Acute Care Surgery |
| Walker | Racial-ethnic variations in paid and unpaid caregiving: Findings among persons with traumatic spinal cord injury | 2015 | Disability and Health Journal |
| Walsh | Ethnic disparities in recovery following distal radial fracture | 2010 | Journal of Bone and Joint Surgery |
| Wandling | Geographic disparities in access to urban trauma care: defining the problem and identifying a solution for gunshot wound victims in Chicago | 2016 | American Journal of Surgery |
| Watson-Thompson | Supporting a community-based participatory evaluation approach to violence prevention in Kansas City | 2013 | Journal of Prevention & Intervention in the Community |
| Weber | Racial Odds for Amputation Ratio in Traumatic Lower Extremity Fractures | 2011 | Journal of Trauma-Injury Infection and Critical Care |
| Wei F | Gender Difference in Falls among Adults Treated in Emergency Departments and Outpatient Clinics | 2014 | Journal of Gerontology and Geriatric Research |
| Weidauer | Do Sex Differences Exist in Rates of Falls and Fractures in Hutterite, Rural, and Nonrural Populations, Aged 20 to 66 Years? | 2015 | Clinical Orthopedic and Related Research |
| Wen H | Racial Differences in Weight Gain: A 5-Year Longitudinal Study of Persons With Spinal Cord Injury | 2017 | Archives of Physical Medicine and Rehabilitation |
| West | Disparities in motor vehicle-related deaths among females—United States, 2005-2009 | 2013 | Journal of Women's Health |
| West et al | Tribal motor vehicle injury prevention programs for reducing disparities motor vehicle-related injuries | 2014 | MMWR Morbidity and Mortality Weekly Report |
| Wexler | Creating a Community of Practice to Prevent Suicide Through Multiple Channels: Describing the Theoretical Foundations and Structured Learning of PC CARES | 2016 | International Quarterly of Community Health Education |
| Weygandt et al | Disparities in mortality after blunt injury: does insurance type matter? | 2012 | Journal of Surgical Research |
| Whaley | The Relationship Between Gender Equality and Rates of Inter- and Intra-Sexual Lethal Violence: An Exploration of Functional Form | 2013 | Justice Quarterly |
| Wheeler | Immigrants as Crime Victims: Experiences of Personal Nonfatal Victimization | 2010 | American Journal of Industrial Medicine |
| Whitfield | American Indians/Native Alaskans With Traumatic Brain Injury Examining the Impairments of Traumatic Brain Injury, Disparities in Service Provision, and Employment Outcomes | 2008 | Rehabilitation Counseling Bulletin |
| Widome | Measuring Neighborhood Connection and the Association with Violence in Young Adolescents | 2008 | Journal of Adolescent Health |
| Will | Tweens at risk: Examining car safety practices in four economically disadvantaged urban elementary schools in Virginia | 2013 | Journal of Safety Research |
| Williams | Ethnic Differences in Posttraumatic Stress Disorder After Musculoskeletal Trauma | 2008 | Journal of Trauma-Injury Infection and Critical Care |
| Williamson | Trajectories of Life Satisfaction Over the First 10 Years After Traumatic Brain Injury: Race, Gender, and Functional Ability | 2016 | Journal of Head Trauma Rehabilitation |
| Wilson S | Identifying disparity in emergency department length of stay and admission likelihood | 2016 | World Journal of Emergency Medicine |
| Wolff | Racial and Ethnic Disparities in Types and Sources of Victimization Inside Prison | 2008 | Prison Journal |
| Wolinsky | Does insurance status affect continuity of care for ambulatory patients with operative fractures | 2011 | Journal of Bone and Joint Surgery |
| Wong | Ethnic differences in risk factors for suicide among American high school students, 2009: The vulnerability of multiracial and Pacific Islander adolescents | 2012 | Archives of Suicide Research |
| Worden | Does insurance status alter short-term outcomes if a standardized fracture treatment pathway is utilized? | 2015 | Current Orthopaedic Practice |
| Wren et al | Racial disparities in fracture risk between white and nonwhite children in the United States | 2012 | The Journal of Pediatrics |
| Wu | Homicide Victimization in California: An Asian and Non-Asian Comparison | 2008 | Violence and Victims |
| Xie | The Effects of Multiple Dimensions of Residential Segregation on Black and Hispanic Homicide Victimization | 2010 | Journal of Quantitative Criminology |
| Yaghoubian et al | Are there racial disparities in the use of restraints and outcomes after motor vehicle collisions in Los Angeles County? | 2011 | Journal of Pediatric Surgery |
| Yard | An epidemiologic comparison of injuries presenting to a pediatric emergency department and local urgent care facilities | 2009 | Journal of Safety Research |
| Young | RACIAL DIFFERENCES IN RECEIVING MORPHINE AMONG PREHOSPITAL PATIENTS WITH BLUNT TRAUMA | 2013 | Journal of Emergency Medicine |
| Yuan | Impact of Safety Net Hospitals in the Care of the Hand-Injured Patient: A National Perspective | 2016 | Agency for Healthcare Research Quality |
| Yun | High Risk Behaviors but Low Injury-Related Mortality Among Hispanic Teens in Missouri | 2016 | Public Health Reports |
| Zarzaur | A Population-Based Analysis of Neighborhood Socioeconomic Status and Injury Admission Rates and In-Hospital Mortality | 2010 | Journal of the American College of Surgeons |
| Zaykowski | Racial disparities in hate crime reporting | 2010 | Violence and Victims |
| Zebib | Geo-demographics of gunshot wound injuries in Miami-Dade county, 2002-2012 | 2017 | BMC Public Health |
| Zelle BA | Ethnic differences in patients' perceptions towards isolated orthopedic injuries: a pilot study | 2017 | Pilot and Feasibility Studies |
| Zhange Lin | Disparity in surveillance of nonfatal motor vehicle crash injuries | 2013 | Traffic Injury Prevention |
| Zimmerman | Do Age Effects on Youth Secondary Exposure to Violence Vary across Social Context? | 2015 | Justice Quarterly |
| Zimmerman | The Covariates of Parent and Youth Reporting Differences on Youth Secondary Exposure to Community Violence | 2014 | Journal of Youth and Adolescence |
| Zimmerman | Examining gendered pathways in the causal chain linking neighborhood navigational strategies and unstructured socializing to adolescent violent offending | 2016 | Journal of Criminal Justice |
| Zimmerman | Investigating the Role of Neighborhood Youth Organizations in Preventing Adolescent Violent Offending: Evidence from Chicago | 2015 | Journal of Quantitative Criminology |
| Zimmerman GM | Gender differences in the effects of parental underestimation of youths' secondary exposure to community violence | 2013 | Journal of Youth and Adolescence |
| Zogg | Implications of the Patient Protection and Affordable Care Act on Insurance Coverage and Rehabilitation Use Among Young Adult Trauma Patients | 2016 | JAMA Surgery |
| Zook | RACIAL DIFFERENCES IN PEDIATRIC EMERGENCY DEPARTMENT TRIAGE SCORES | 2016 | American Journal of Industrial Medicine |
